# Effectiveness and cost-effectiveness of Self-Help Plus (SH+) for preventing mental disorders in refugees and asylum seekers in Europe and Turkey: study protocols for two randomised controlled trials

**DOI:** 10.1136/bmjopen-2019-030259

**Published:** 2019-05-14

**Authors:** Marianna Purgato, Kenneth Carswell, Ceren Acarturk, Teresa Au, Sena Akbai, Minna Anttila, Josef Baumgartner, Della Bailey, Massimo Biondi, Martha Bird, Rachel Churchill, Sevde Eskici, Louise Juul Hansen, Paul Heron, Zeynep Ilkkursun, Reinhold Kilian, Markus Koesters, Tella Lantta, Michela Nosè, Giovanni Ostuzzi, Davide Papola, Mariana Popa, Marit Sijbrandij, Lorenzo Tarsitani, Federico Tedeschi, Giulia Turrini, Ersin Uygun, Maritta Anneli Välimäki, Johannes Wancata, Ross White, Elisa Zanini, Pim Cuijpers, Corrado Barbui, Mark Van Ommeren

**Affiliations:** 1 Department of Neurosciences, Biomedicine and Movement Sciences, University of Verona, Verona, Italy; 2 Cochrane Global Mental Health, Verona, Italy; 3 Department of Mental Health & Substance Abuse, World Health Organisation, Geneve, Switzerland; 4 Istanbul Sehir Universitesi, Istanbul, Turkey; 5 Department of Psychology, Istanbul Sehir Universitesi, Istanbul, Turkey; 6 Department of Nursing Science, University of Turku, Turku, Finland; 7 Department of Psychiatry and Psychotherapy, Medical University of Vienna, Wien, Austria; 8 Health Sciences, University of York, York, UK; 9 Department of Human Neurosciences, University of Rome La Sapienza, Roma, Lazio, Italy; 10 IFRC Reference Centre for Psychosocial Support, Danish Red Cross, Copenhagen, Denmark; 11 University of York, York, UK; 12 Department of Health Sciences, University of York, York, UK; 13 Department of Psychiatry II, Ulm University, Ulm, Germany; 14 Department of Nursing Science, Turun Yliopisto, Turku, Finland; 15 Institute of Life and Human Sciences, University of Liverpool, Liverpool, UK; 16 Vrije Universiteit Amsterdam, Amsterdam, The Netherlands; 17 Trauma and Disaster Mental Health, Istanbul Bilgi Universitesi, Istanbul, Turkey; 18 School of Nursing, Hong Kong Polytechnic University, Kowloon, Hong Kong; 19 Department of Psychiatry and Psychotherapy, Division of Social Psychiatry, Medical University of Vienna, Vienna, Austria; 20 Department of Clinical Psychology, VU University Amsterdam, Amsterdam, The Netherlands; 21 Department of Mental Health and Substance Dependence, World Health Organisation, Geneva, Switzerland

**Keywords:** randomized controlled trials, psychosocial interventions, asylum seekers, refugees, global mental health

## Abstract

**Introduction:**

This article describes two randomised controlled trials that will evaluate the effectiveness and cost-effectiveness of Self-Help Plus (SH+), a group self-help intervention developed by the WHO to reduce distress. In these trials SH+ is being tested as a preventative intervention to lower the incidence of mental disorders in asylum seekers and refugees with psychological distress resettled in Europe and Turkey.

**Methods and analysis:**

Two prospective, multicentre, randomised, rater-blinded, parallel-group studies will follow participants over a period of 12 months. One trial will be conducted in Europe and one in Turkey. In each trial, 600 asylum seekers and refugees screening positive on the General Health Questionnaire (≥3), but without a formal diagnosis of any mental disorders according to the Mini International Neuropsychiatric Interview, will be randomly allocated to SH+or to enhanced treatment-as-usual. The primary outcome will be a lower incidence of mental disorders at 6 month follow-up. Secondary outcomes will include the evaluation of psychological symptoms, functioning, well-being, treatment acceptability and indicators of intervention cost-effectiveness.

**Ethics and dissemination:**

The two trials received ethical clearance from the local Ethics Committees of the participating sites (seven sites), as well as from the WHO Ethics Committee. All participants will provide informed consent before screening and before study inclusion (a two-step procedure). The results of the trials will be disseminated in agreement with a dissemination plan that includes publication(s) in peer-reviewed journals and presentations at relevant national and international conferences and meetings.

**Trials registration numbers:**

NCT03571347, NCT03587896.

**Strengths and limitations of this study**

- Interventions for preventing mental disorders are a public health priority.
- Evaluating the effectiveness of a low-intensity group self-help intervention is particularly relevant in emergency and limited-resource settings.
- Conducting research with populations in transit is challenging, as participants might not be traceable at follow-up and may not be interested in participating in clinical studies.
- The possibility of contamination between the experimental and control arms cannot be excluded, as randomisation will occur at the individual level.

## Introduction

### Background and rationale

The number of people seeking refugee status in European and bordering countries has progressively increased in recent years, driven by the wars in Syria, Afghanistan and Iraq, alongside conflicts and instability in Afghanistan and elsewhere.^1–3^ These populations face numerous challenges before, during and after migration.^4–8^ Refugees and asylum seekers are exposed to multiple stressors in their native countries (eg, war-related experiences, violence, destructions of their homes). They are also exposed to many stressors during migration.^6 9^ After arriving in host countries, they may then face additional challenges and perceived discrimination due to language barriers, loss of family and community support, poverty, lack of access to social, educational and healthcare institutions and uncertain asylum application procedures.^10 11^

These factors place refugees and asylum seekers at considerable risk for developing common mental disorders, including post-traumatic stress disorder (PTSD), anxiety disorders and depressive disorders and other forms of disabling psychological distress.^12 13^

There is a growing body of research demonstrating the effectiveness of psychological therapies for refugees and asylum seekers.^12 14^ A recently-published umbrella review of systematic reviews on this topic showed encouraging results in terms of reduction of depression, PTSD and other anxiety symptoms.^12^ However, treatments typically require extensive training and supervision, and sufficient time to be delivered in full (eg, up to 12 weeks). Additionally, most interventions targeted the symptoms of mental disorders, without a specific focus on prevention.^8 12 15–17^

Recently, the WHO has developed a novel, low-intensity five-session self-help intervention for managing stress and coping with adversity, which is designed to be delivered by non-specialist facilitators and does not require extensive training.^18^ This psychosocial intervention, called Self-Help Plus (SH+), is intended to help people with and without mental disorders to cope with distress stemming from diverse types of adversity. SH+ has already been tested in small pilot studies^19 20^ and a large positive randomised controlled trial (RCT) with South Sudanese female refugees in Uganda.^19 20^ SH+ has not been tested as a preventive intervention.

In order to fill this gap, two RCTs were designed to test SH+ as an indicated preventive intervention with asylum seekers and refugees resettled in European countries and in Turkey. The choice of conducting two separate trials is mainly due to the type of setting and the different travelling experiences of asylum seekers and refugees.

### Aim and objectives

The overall aim of these RCTs is to evaluate the effectiveness and cost-effectiveness of SH+ as an indicated preventive intervention in asylum seekers and refugees with psychological distress resettled in six sites of five European countries (Italy, Austria, Germany, Finland and two sites in the UK), and in Turkey, as compared with enhanced treatment as usual (ETAU). The primary objective is the reduction in the incidence of any mental disorder at 6 month follow-up. Secondary objectives are the evaluation of psychological symptoms, functioning, well-being, treatment acceptability and economical outcomes.

### Study hypotheses

1. SH+ will be superior to ETAU in preventing the onset of any mental disorder at 6 month follow-up.
2. Compared with the ETAU group, asylum seekers and refugees in the SH+ intervention arm will report an improvement in depression, anxiety and PTSD symptoms, psychosocial well-being and improved levels of psychological functioning.
3. Asylum seekers and refugees in the SH+ intervention arm will incur lower healthcare costs compared with the ETAU group.

## Methods and analyses

**Figure 1:**
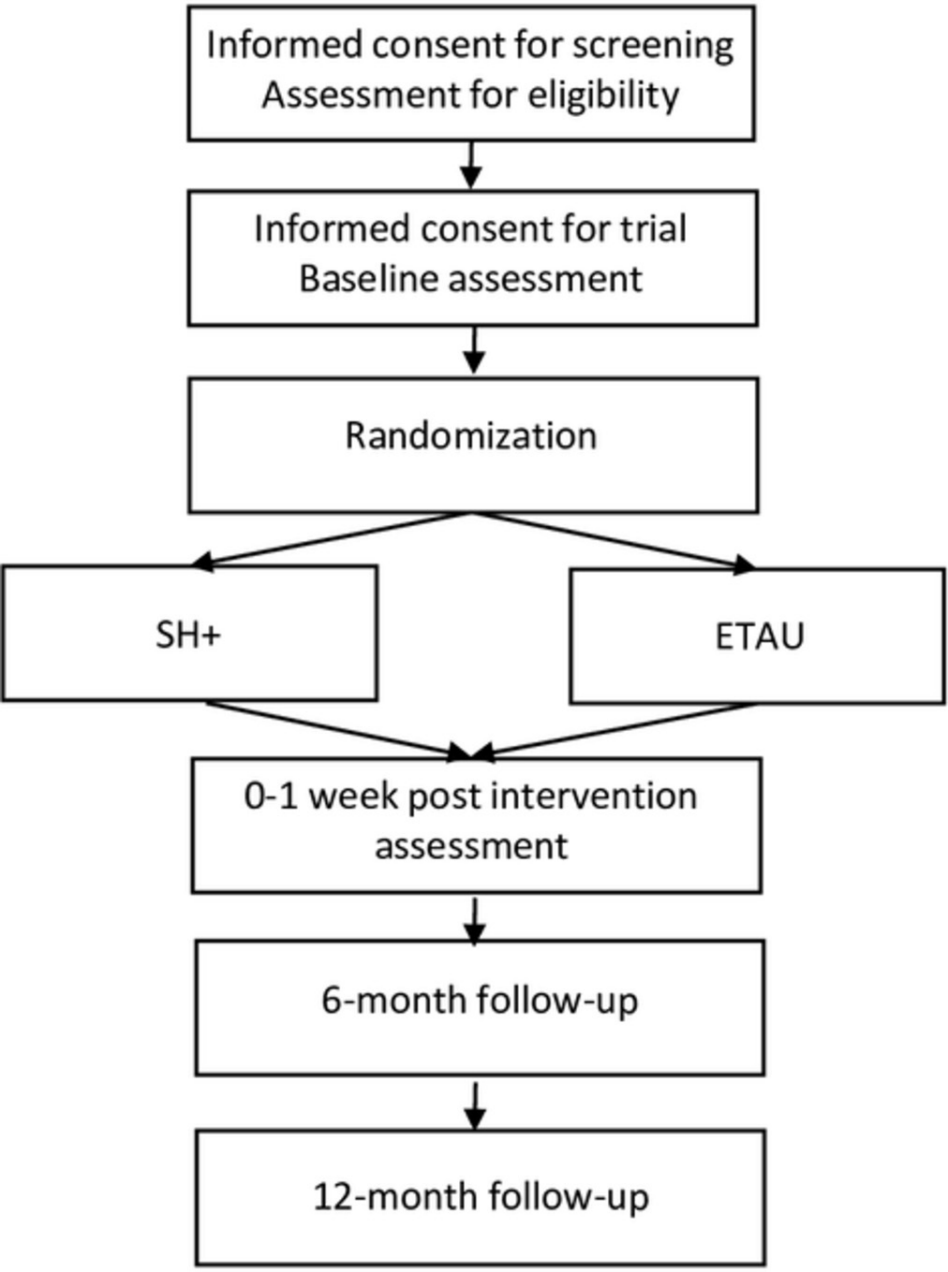
RCT flow diagram. ETAU, enhanced treatment as usual; RCT, randomised controlled trial; SH+, Self-Help Plus.

These are two prospective, multicentre, randomised, parallel-group studies that will follow participants over a period of 12 months. Figure 1 provides a flow-chart of the RCT’s process.

Refugees and asylum seekers with psychological distress as assessed by the General Health Questionnaire (GHQ),^21^ but without a mental disorder according to the Mini International Neuropsychiatric Interview (M.I.N.I.)^22^ for the Diagnostic and Statistical Manual of Mental Disorders, fifth edition (DSM-5)^23^ and the International Classification of Diseases, tenth edition (ICD-10) classification of mental and behavioural disorders,^24^ will be randomly assigned to SH+ or ETAU. Participants will be assessed at baseline before randomisation, immediately post-intervention, and at 6 and 12 months of follow-up. The two RCTs will be conducted in accordance to the Consolidated Standards of Reporting Trials statement.^25 26^

### Inclusion criteria

- Age 18 or above;
- Able to speak and understand one of the languages in which SH+ has been adapted: Arabic, Dari, Urdu or English;
- Asylum seeker or refugee or person under temporary protection;
- Presence of psychological distress, as shown by a score of 3 or more on the 12-item GHQ-12;
- Both oral and written informed consent to enter the study.

### Exclusion criteria

- Presence of any mental disorders according to DSM-5 and ICD-10, as shown by a positive M.I.N.I.;
- Acute medical conditions contraindicating study participation, based on the clinical judgement of the healthcare professional or trained paraprofessional who performs the screening;
- Clinical evidence of imminent suicide risk or suicide risk scored as ‘moderate or high’ (or a positive suicidality behaviour disorder) by the M.I.N.I. (section Suicidality);
- Clinical evidence that decision-making capacity is impaired.

Asylum seekers and refugees that are excluded because of a diagnosis of a mental disorder will be advised to seek professional treatment according to a pre-defined protocol.

Two experts giving advice on any ethical issues related to the trials were appointed to be members of an Ethics Advisory Board (EAB). Additionally, a data protection officer was appointed to supervise and report that data collection is carried out according to European and national legislations.

### Characteristics of the intervention

The SH+ programme has been developed by WHO and collaborators working in the humanitarian field. SH+ consists of a pre-recorded audio course, delivered by facilitators in a group setting and complemented with an illustrated self-help book. The potential value of using a psychoeducational course to access hard-to-reach populations has been shown previously.^27^ Evidence for the use of books in interventions is also promising.^28^ Furthermore, research has found that guided self-help programmes produce much better results than ‘pure’ self-help, and the effects produced are similar to face-to-face psychological treatment.^29^ SH+ was designed to be relevant for large segments of adversity-affected populations: it is intended to be transdiagnostical, easily adaptable to different cultures and languages and both meaningful and safe for people with and without mental disorders.

The format of SH+ is innovative in that it seeks to ensure that key intervention components are delivered as intended without the burden of extensive facilitator training. The SH+ programme is based on acceptance and commitment therapy (ACT), a form of cognitive-behavioural therapy, with distinct features.^30^ ACT is based on the concept that ongoing attempts to suppress unwanted thoughts and feelings can paradoxically make these problems worse. Instead, it emphasises learning new ways to accommodate difficult thoughts and feelings, while guiding people to take proactive steps towards living in a way that is consistent with their values. ACT has been shown to be useful for a range of mental health issues^31^ and has been used successfully in a guided self-help format.^32^ SH+ includes ACT techniques that aim to help people cope with stress, respond compassionately to themselves and others and to live in accordance with their values.

The SH+ programme has two components: a pre-recorded course and an illustrated self-help book. Pre-recorded audio material is delivered across five 2 hour sessions to groups of up to 30 people. The audio material imparts key information about stress management and guides participants through individual exercises and small group discussions. To augment the audio recordings, an illustrated self-help book reviews all essential content and concepts.^18^ Written manuals help non-specialist facilitators to conduct the course using the pre-recorded audio.

#### Delivery of SH+

SH+ will be delivered by briefly-trained, non-specialist facilitators with a refugee or migrant background. SH+ trainers will be academics and/or mental healthcare professionals. Before the recruitment period, SH+ trainers received specific training on the SH+ intervention from WHO.

SH+ has been adapted into Arabic, Dari and Urdu according to the WHO Department of Mental Health and Substance Abuse draft protocol for adapting psychological interventions for common mental health problems. An English version is also available, which has been translated into the other languages with a focus on ensuring the translated version text and images are acceptable, understandable and relevant to the population. Close attention was paid to the form of language (eg, colloquial) and tone (eg, warm and caring) so that it may be fully suitable for a pre-recorded audio psychological intervention. The SH+ audio and book were reviewed and revised by native speakers with diverse backgrounds.

#### Enhanced treatment as usual

Control arm participants will receive routine social support and/or care following local regulations. Additionally, they will receive baseline and follow-up assessments according to the study schedule; information about freely available health and social services, and community networks that provide support to refugees and asylum seekers.

### Primary and secondary outcomes

Outcome measures and time points are detailed in table 1. The primary outcome will be the incidence of any mental disorders measured by the M.I.N.I. at 6 month follow-up. All other measures will be secondary outcomes.

**Table 1:** Outcome measures and time points

Concept	Screening	Baseline		Post- intervention	6 month follow-up	12 month follow-up
	Informed consent	Informed consent	Randomisation			
**Mental capacity**	Mental capacity form	Mental capacity form		Mental capacity form	Mental capacity form	Mental capacity form
**Psychological distress**	GHQ-12			GHQ-12	GHQ-12	GHQ-12
**Psychiatric diagnosis**	M.I.N.I.			M.I.N.I.	M.I.N.I.	M.I.N.I.
**Socio-demographical and migration data**		Recruitment form				
**Symptoms of PTSD**		PCL-5		PCL-5	PCL-5	PCL-5
**Depressive symptoms**		PHQ-9		PHQ-9	PHQ-9	PHQ-9
**Adverse life events**		HTQ –Part A/1				
**Subjective well-being**		WHO-5 Well-being index		WHO-5 Well-being index	WHO-5 Well-being index	WHO-5 Well-being index
**Health-related quality of life**		EQ-5D-3L			EQ-5D-3L	EQ-5D-3L
**Self-defined psychosocial goals**		PSYCHLOPS (pre-int. version)		PSYCHLOPS (post-int. version)	PSYCHLOPS (post-int. version)	PSYCHLOPS (post-int. version)
**Post migration stress difficulties**				PMLD	PMLD	PMLD
**Psychological functioning**		WHODAS 2.0		WHODAS 2.0	WHODAS 2.0	WHODAS 2.0
**Cost-effectiveness**		CSSRI-EU			CSSRI-EU	CSSRI-EU
**Adverse events**	**Adverse events form**					

CSSRI-EU, Client Socio-Demographic and Service Receipt Inventory, European Version; DSM-5, Diagnostic and Statistical Manual of Mental Disorders, fifth edition; GHQ-12, General Health Questionnaire-12 items; HTQ, Harvard Trauma Questionnaire; int., intervention; MINI, Mini International Neuropsychiatric Interview; PCL-5, PTSD Checklist for DSM-5; PHQ-9, Patient Health Questionnaire–9 items; PMLD, post-migration living difficulties; PSYCHLOPS, Psychological Outcome Profiles Instrument; PTSD, Post-Traumatic Stress Disorder; WHO-5, World Health Organization–5 items; EQ-5D-3L, Three-level version of EuroQol Group; WHODAS, World Health Organization Disability Assessment Schedule.

### Randomisation

Randomisation will occur at the individual level and will be stratified by each recruiting centre. To avoid contamination, only one person per household will be randomised. Randomisation will be centralised and coordinated by the WHO Collaborating Centre of the University of Verona. Eligible participants will be randomly assigned to one of the two groups with an equal probability of assignment to each group (allocation ratio 1:1). The randomisation schedule will be generated using the web-based software Castor Electronic Data Capture.^33^ This electronic tool employs a variable block randomisation method, in order to allocate groups randomly permuted in blocks of unequal size. The site investigators will not know the block size and will not be able to access the randomisation list. The randomisation list will be accessible only to the data manager. In addition, the web-based software will allow random allocation only after the main information on the enrolled participant is entered, on verification of the inclusion criteria. After random allocation, the software will produce a unique identification number for each participant. In accordance with the Declaration of Helsinki,^34^ the participants’ confidentiality will be preserved at all times and the contents of the recruitment and follow-up forms will not be disclosed to any third party.

### Masking

Masking participants and facilitators about the intervention status will be impossible, due to the nature of the intervention. However, investigators evaluating primary and secondary outcomes post-intervention and at 6 and 12 month follow-up, and the statistician performing all analyses, will be masked to the participants’ allocation status. Healthcare professionals and cultural mediators involved in the assessments will be instructed on how to perform follow-up assessments in order to preserve effective masking.

### Sample size and power calculations

On the basis of data extrapolated from prevention trials, an event rate (diagnosis of any mental disorders) of around 15% at 6 months (primary outcome) is expected.^35 36^ However, these prevention trials were conducted in unselected populations that were not exposed to migration stressors. By contrast, refugees and asylum seekers are exposed to stressors that are associated with increased rates of mental disorders.^8^ On these grounds, we anticipate an incidence rate of mental disorders of 25% at 6 months in this population. It is hypothesised that the provision of the SH+ programme will show a clinically significant advantage by producing a between-groups absolute difference of 10%. A sample size of 500 participants per trial, achieves 80% power for a 0.05 level of significance between the two proportions of people diagnosed with mental disorders at 6 months. Assuming that a relevant proportion of asylum seekers and refugees might be lost at study endpoint (due to the specific characteristics of this population), the final sample size will be of 600 participants (300 in each group) for the European trial and 600 participants (300 in each group) for the Turkish trial. We anticipate that we will screen around 1800 to 2000 people to have 600 eligible participants.

### Adverse events reporting

Serious adverse events and other adverse events reported spontaneously by the participants or observed by the research staff will be recorded on a specifically developed form. Data on the relationship with the study intervention, the action taken regarding intervention and the outcome of the adverse event, will be collected. An event is considered a potential adverse reaction if it is an undesirable experience occurring to a participant during the study, whether or not it is considered related to the research procedure. This definition includes all aspects of mental health and psychological functioning, but also any other undesirable experiences. The EAB will review spontaneously reported serious adverse reactions (eg, suicide attempts) within 48 hours, while general adverse reactions will be reviewed by the EAB in regular meetings.

### Data management and statistical analysis

#### Statistical analysis

##### General approach

The statistical analysis will be masked. All analyses will be performed using Stata/SE, Release 14.2.

Two data locks will occur during the study. The first will happen 6 months after the end of the 12 month enrolment period, when information on the primary outcome and on short-term secondary outcomes will be available for all the participants. The second will happen 12 months after the end of the 12 month enrolment period. All primary and secondary analyses will be performed on an intention-to-treat (ITT) basis. The ITT population will consist of all participants randomly assigned to the competing intervention strategies, and with data on the baseline assessment available. In order to check the robustness of results, the primary outcome will be additionally analysed using a per protocol (PP) approach, that will include only SH+ participants who attended at least three sessions. The analysis of the PP population will be used for confirmatory purposes only. If less than 5% of participants do not receive the allocated intervention according to the study protocol, the PP analysis will not be performed. All analyses reaching statistical significance will be replicated for each recruiting centre and for each target language separately.

##### Analysis of the primary outcome

The proportion of participants with a diagnosis of any mental disorders at 6 months follow-up will be compared between the two groups using a chi-square test (primary analysis). A multivariate analysis (secondary analysis) will be performed through a Poisson regression model, with robust error variance,^34^ to estimate relative risks directly and to explore the potential confounding effect of prognostic factors, and the interactions with treatment.

##### Analysis of the secondary outcomes

The proportion of participants with a diagnosis of any mental disorder post-intervention and at 12 months of follow-up will be compared between the two groups using a X^2^ test. A multivariate analysis will be performed using a Poisson regression model, with a robust error variance, to estimate relative risks directly and to explore the potential confounding effect of prognostic factors, and the interactions with treatment.^37^

##### Analysis of GHQ-12, WHODAS 2.0, PHQ-9, WHO-5 Well-being index, PSYCHLOPS, PCL-5

For each questionnaire, in case of missing items, the corrected item mean substitution method,^38^ will be used (ie, the item mean across participants weighted by the subject’s mean of completed items), using information from subjects belonging to the same treatment arm for the same follow-up time. The hypothesis that the experimental intervention has no effect on GHQ-12, World Health Organisation Disability Assessment Schedule (WHODAS) 2.0, Patient Health Questionnaire–9 items (PHQ-9), WHO-5 Well-being index, Psychological Outcome Profiles Instrument (PSYCHLOPS), PTSD Checklist for DSM-5 (PCL-5) scores will be tested by performing seemingly unrelated regression,^39^ for each time point, controlling for baseline values. In case of joint statistical significance of the coefficients related to treatment status, the effect of treatment on each score will be evaluated through a mixed analysis of covariance (ANCOVA), controlling for the value at baseline. After the last data lock, ANCOVA with robust standard errors will be performed for each scale, using the values at post-intervention, and at 6 and 12 months follow-up, controlling for the value at baseline. In case of joint statistical significance of the coefficients related to treatment status, the analysis will be repeated for each outcome/time combination. Such approaches (assessing the global significance of all time locks for each scale, without imputing missing values) will allow to have a robustness check to results obtained after each time point. As a further sensitivity check, multivariate analyses will be performed for each scale to take confounding factors into account, again including the baseline value as a covariate.

The proportion of participants leaving the study early will be compared between the two groups using a X^2^ test (or the Fisher exact test, where appropriate). A multivariate analysis will be performed using a Poisson regression model, with a robust error variance, to estimate relative risks directly and to explore the potential confounding effect of prognostic factors, and the interactions with treatment.^37^

### Cost-effectiveness analysis

Incremental cost-effectiveness analyses (ICEA) will be conducted from the health and social care system perspective and from the societal perspective by means of the net-benefit approach.^40^ For the five European countries the ICEA will be conducted from the perspective of the UK healthcare system and for Turkey from the perspective of the Turkish healthcare system. Direct costs including formal medical and psychiatric healthcare, psychosocial care, legal services and informal services will be used for ICEA from the health and social care system (payer) perspective. Total costs will be used for ICEA from the societal perspective. Comprehensive use of annual health and social care resources will be assessed by means of completing the adapted Client Socio-Demographic and Service Receipt Inventory, European Version. Information on the unit-costs of the used services for the European countries will be gained by the cost compilation for the UK health and social care system.^41^ For Turkey, price lists and other sources will be used to identify unit costs. Costs of informal services will be estimated for both studies on the basis of average salaries per hour. Total direct costs of service use will be calculated by multiplying service units with unit costs. Annual total costs of mental illness will be estimated on the basis of two retrospective assessments of 6 month service use at 6 and 12 month follow-up. Productivity losses will be estimated for participants with work permissions by means of the human capital approach^42^ on the basis of group-specific average salaries. Costs for the European countries will be calculated in 2017 UK £. Costs for Turkey will be calculated in Turkish Lira. Total costs of health and social care service use will be investigated with regard to the cost driving effects of asylum seekers and refugees characteristics and the impact of different levels of coverage, different macroeconomical, social and healthcare indicators by estimating regression based cost functions.^43^ Incremental net monetary benefit (NMB) will be computed by regressing the NMB on study group.^44^ Results of ICEA for the European countries will be interpreted according to UK marginal willingness to pay (MWTP) thresholds recommended by the National Institute for Health and Care Excellence (NICE).^45^ Results for Turkey will be interpreted according to the three times gross domestic product per capita threshold^46^ and alternatively by country-specific thresholds provided by Woods *et al*.^47^ For international comparison all cost data and results of ICEA will be converted into € and US$ adjusted for power purchasing parities.

### Patient and public involvement

Participants have not been involved in the design of the study. However, SH+ facilitators delivering the intervention will be refugees or community members with a refugee or migrant background or with the same/similar culture of participants. They will have some prior experience in health or social or community work or volunteering. The choice of asylum seekers’ and refugees’ country of origin was made in accordance with a situational analysis on size of refugee populations within European countries and Turkey, as well as feasibility issues.

### Ethics and dissemination

It is essential to conduct this research because rigorous evidence needs to be collected on the effectiveness of the SH+ intervention, which has been specifically developed for vulnerable populations. Additionally, it will provide valuable information about optimal adaptation strategies and aspects to consider when scaling up the intervention in diverse contexts.

The trials will be conducted according to globally accepted standards of good clinical practice (as defined in the ICH E6 Guideline for Good Clinical Practice, 1 May 1996), in agreement with the Declaration of Helsinki and in keeping with local regulations.

Dissemination includes a publication plan agreed by all members of the consortium that regulates academic articles and conference presentations. On the basis of positive results from research trials of SH+, the SH+ package will be disseminated by WHO in multiple languages. Any protocol modifications will be submitted to local Ethics Committess for approval.

## Discussion

This article presents the main characteristics of two RCTs, which will assess the effectiveness and cost-effectiveness of a low-intensity self-help intervention to prevent mental disorders among distressed refugees and asylum seekers. The concept of low-intensity implies that fewer resources are used than in more complex, conventional psychosocial treatments. SH+ may be delivered by facilitators without formal mental health professional training, employs low-cost technologies, such as audio recordings and an illustrated book, has only five sessions and may be delivered to groups of up to 30 participants. The group setting has the advantage of being scalable and optimising resource use, but it has limited scope for in-depth work with individuals, and participants exposed to traumatic experiences might be concerned about interacting with others in a group space.

If positive results from these trials are achieved, WHO may adopt and disseminate SH+ as a preventive intervention in multiple languages.

A number of characteristics of the design of these two trials may hamper or facilitate their implementation. First, the study was designed as two trials with an almost identical design, with one conducted in European countries and one in Turkey. We reasoned that even though the population that will be recruited in Turkey shares important characteristics with the population in European countries, in terms of pre-migration, migration and post migration stressors,^5 9 48^ the resettlement context is likely to be different. This may be relevant for a number of aspects, including the definition of the control group, which receives treatment as usual enhanced by referral mechanisms to social organisations and healthcare facilities. While these mechanisms may show some similarities across different European countries, the same may not apply for Turkey. The resettlement context may additionally be relevant for the capacity of research staff to follow participants, as this aspect may be substantially affected by the mobility of the population under study.

Second, a focus on prevention, rather than treatment, is challenging. In practical terms, it means recruiting participants showing some psychological distress but not a mental disorder. This may represent an obstacle to recruitment, as at study entry the administration of the M.I.N.I., which is of paramount relevance to exclude those with a disorder, requires time and training. Additionally, since participants who screen positive cannot be included, we anticipate the need to screen hundreds of potential participants to reach the target sample size. Another aspect related to the recruitment of participants without a disorder is that those eligible for inclusion will clearly be less distressed as compared with those suffering from a mental disorder. Therefore, eligible participants may be less likely to agree to be included, as they may feel that dealing with their psychological distress may not be as relevant as dealing with other social or health aspects. Participants with a mental disorder, by contrast, may recognise that dealing with psychological problems is a high priority and a pre-requisite for optimal social functioning. This may be another obstacle for effective recruitment.

Despite these potential challenges, establishing the effectiveness of preventive psychosocial interventions is a public health priority, as it may help large population groups over short periods of time. Preventive psychosocial interventions must be feasible, sustainable and cost-effective. SH+, which is based on self-help approaches, may have particular advantages for populations with limited access to social and healthcare services, and with high levels of need.

## Conclusion

This project aims to generate a strong evidence-base for preventative aspects of SH+, and to create a scientific framework for adapting and equipping social and healthcare systems in countries inside and outside Europe with such a low intensity intervention. The delivery format of SH+ is innovative, as it requires a short training of non-professional facilitators, and reduced supervision, and fidelity is assured by the pre-recorded audio and the book. Moreover, the intervention has been culturally adapted according to a WHO protocol and cultural adaptation will be extended to other population groups after publication of the SH+ programme.

## Supplementary Material

Reviewer comments

Author's manuscript
