# Supplementary material for: Effectiveness and cost-effectiveness of Self-Help Plus (SH+) for preventing mental disorders in refugees and asylum seekers in Europe and Turkey: study protocols for two randomised controlled trials
Source: BMJ Open. 2019 May 14;9(5):e030259. doi: 10.1136/bmjopen-2019-030259 (PMC6530324; doi:10.1136/bmjopen-2019-030259)
Supplement: Author's manuscript [file bmjopen-2019-030259.draft_revisions.pdf]

BMJ Open is committed to open peer review. As part of this commitment we make the peer review history of every article we publish publicly available.

When an article is published we post the peer reviewers' comments and the authors' responses online. We also post the versions of the paper that were used during peer review. These are the versions that the peer review comments apply to.

The versions of the paper that follow are the versions that were submitted during the peer review process. They are not the versions of record or the final published versions. They should not be cited or distributed as the published version of this manuscript.

BMJ Open is an open access journal and the full, final, typeset and author-corrected version of record of the manuscript is available on our site with no access controls, subscription charges or pay-per-view fees (http://bmjopen.bmj.com).

If you have any questions on BMJ Open's open peer review process please email info.bmjopen@bmj.com

BMJ Open

Effectiveness and cost-effectiveness of Self Help Plus (SH+) for preventing mental disorders in refugees and asylum seekers in Europe and Turkey: study protocols for two randomized controlled trials

Journal:	*BMJ Open*
Manuscript ID	bmjopen-2019-030259
Article Type:	Protocol
Date Submitted by the Author:	07-Mar-2019

Complete List of Authors:	Purgato, Marianna; University of Verona, Department of Neurosciences, Biomedicine and Movement Sciences; Cochrane Global Mental Health<br>Carswell, Kenneth; World Health Organization, Department of Mental Health & Substance Abuse<br>Acarturk, Ceren; Istanbul Sehir Universitesi,<br>Au, Teresa; World Health Organization, Department of Mental Health & Substance Abuse<br>Akbai, Sena; Istanbul Sehir Universitesi, Department of Psychology<br>Anttila, Minna; University of Turku, Department of Nursing Science<br>Baumgartner, Josef; Medical University of Vienna, Department of Psychiatry and Psychotherapy<br>Bailey, Della; University of York, Health Sciences<br>Biondi, Massimo; University of Rome La Sapienza, Department of Human Neurosciences<br>Bird, Martha; Danish Red Cross, IFRC Reference Centre for Psychosocial Support<br>Churchill, R; University of York,<br>Eskici, Sevde; Istanbul Sehir Universitesi, Department of Psychology<br>Hansen, Louise; Danish Red Cross, IFRC Reference Centre for Psychosocial Support<br>Heron, Paul; University of York, Department of Health Sciences<br>Ilkkursun, Zeynep; Istanbul Sehir Universitesi, Department of Psychology<br>Kilian, Reinhold; Ulm University, Department of Psychiatry II<br>Koesters, Markus; Ulm University, Department of Psychiatry II; Cochrane Global Mental Health<br>Lantta, Tella; Turun Yliopisto, Department of Nursing Science<br>Nosè, Michela; University of Verona, Department of Neurosciences, Biomedicine and Movement Sciences; Cochrane Global Mental Health<br>Ostuzzi, Giovanni; University of Verona, Department of Neurosciences, Biomedicine and Movement Sciences; Cochrane Global Mental Health<br>Papola, Davide; University of Verona, Department of Neurosciences, Biomedicine and Movement Sciences; Cochrane Global Mental Health<br>Popa, Mariana; University of Liverpool, Institute of Life and Human Sciences<br>Sijbrandij, Marit; Vrije Universiteit Amsterdam,<br>Tarsitani, Lorenzo; University of Rome La Sapienza, Department of Human Neurosciences

Tedeschi, Federico; University of Verona, Department of Neurosciences, Biomedicine and Movement Sciences
Turrini, Giulia; University of Verona, Department of Neurosciences, Biomedicine and Movement Sciences; Cochrane Global Mental Health
Uygun, Ersin; Istanbul Bilgi Universitesi, Trauma and Disaster Mental Health
Välimäki, Maritta; University of Turku, Department of Nursing Science; Hong Kong Polytechnic University, School of Nursing
Wancata, Johannes; Medical University of Vienna, Department of Psychiatry and Psychotherapy, Division of Social Psychiatry
White, Ross; University of Liverpool, Institute of Life and Human Sciences
Zanini, Elisa; University of Verona, Department of Neurosciences, Biomedicine and Movement Sciences
Cuijpers, Pim; VU University Amsterdam, Department of Clinical Psychology
Barbui, Corrado; University of Verona, Department of Neurosciences, Biomedicine and Movement Sciences; Cochrane Global Mental Health
Van Ommeren, Mark; World Health Organization, Dep Mental Health and Substance Dependence

Keywords: randomized controlled trials, psychosocial interventions, asylum seekers, refugees, global mental health

SCHOLARONE™ Manuscripts

Note from the Editors: Instructions for reviewers of study protocols

Since launching in 2011, BMJ Open has published study protocols for planned or ongoing research studies. If data collection is complete, we will not consider the manuscript.

Publishing study protocols enables researchers and funding bodies to stay up to date in their fields by providing exposure to research activity that may not otherwise be widely publicised. This can help prevent unnecessary duplication of work and will hopefully enable collaboration. Publishing protocols in full also makes available more information than is currently required by trial registries and increases transparency, making it easier for others (editors, reviewers and readers) to see and understand any deviations from the protocol that occur during the conduct of the study.

The scientific integrity and the credibility of the study data depend substantially on the study design and methodology, which is why the study protocol requires a thorough peer-review.

BMJ Open will consider for publication protocols for any study design, including observational studies and systematic reviews.

Some things to keep in mind when reviewing the study protocol:

- Protocol papers should report planned or ongoing studies. The dates of the study should be included in the manuscript.
- Unfortunately we are unable to customize the reviewer report form for study protocols. As such, some of the items (i.e., those pertaining to results) on the form should be scores as Not Applicable (N/A).
- While some baseline data can be presented, there should be no results or conclusions present in the study protocol.
- For studies that are ongoing, it is generally the case that very few changes can be made to the methodology. As such, requests for revisions are generally clarifications for the rationale or details relating to the methods. If there is a major flaw in the study that would prevent a sound interpretation of the data, we would expect the study protocol to be rejected.

For peer review only

Effectiveness and cost-effectiveness of Self Help Plus (SH+) for preventing mental disorders in refugees and asylum seekers in Europe and Turkey: study protocols for two randomized controlled trials

Marianna Purgato[1,2]*, Kenneth Carswell[3], Ceren Acarturk[4], Teresa Au[3], Sena Akbay[4], Minna Anttila[5], Joseph Baumgartner[6], Della Bailey[7], Massimo Biondi[8], Martha Bird[9], Rachel Churchill[10], Sevde Eskici[4], Louise Juul Hansen[9], Paul Heron[7], Zeynep Ilkkursun[4], Reinhold Kilian[11], Markus Koesters[2,11], Tella Lantta[5], Michela Nosè[1,2], Giovanni Ostuzzi[1,2], Davide Papola[1,2], Mariana Popa[12], Marit Sijbrandij[13], Lorenzo Tarsitani[8], Federico Tedeschi[1], Giulia Turrini[1,2], Ersin Uygun[14], Maritta Valimaki[5,15], Johannes Wancata[6], Ross White[12], Elisa Zanini[1], Pim Cuijpers[13], Corrado Barbui[1,2†], Mark van Ommeren[3†]

1. WHO Collaborating Centre for Research and Training in Mental Health and Service Evaluation, Department of Neuroscience, Biomedicine and Movement Sciences, Section of Psychiatry, University of Verona, Verona, Italy.

2. Cochrane Global Mental Health, University of Verona, Verona, Italy.

3. Department of Mental Health & Substance Abuse, World Health Organization, Geneva, Switzerland

4. Department of Psychology, Istanbul Sehir University, Istanbul, Turkey

5. University of Turku, Faculty of Medicine, Department of Nursing Science, Finland

6. Clinical Division of Social Psychiatry, Department of Psychiatry and Psychotherapy, Medical University of Vienna, Vienna, Austria

7. Department of Health Sciences, University of York, York, UK

8. Department of Human Neurosciences, Sapienza University of Rome, Italy

9. Danish Red Cross, IFRC Reference Centre for Psychosocial Support, Copenhagen, Denmark

10. Centre for Reviews and Dissemination, University of York, York, UK

11. Department of Psychiatry II, Ulm University, Ulm, Germany

For peer review only

12. Institute of Life and Human Sciences, University of Liverpool, UK

13. Department of Clinical, Neuro- and Developmental Psychology, Amsterdam Public Health research institute, Vrije Universiteit, Amsterdam, The Netherlands

14. Trauma and Disaster Mental Health, Istanbul Bilgi University, Istanbul, Turkey

15. Hong Kong Polytechnic University, School of Nursing, Hong Hom, Kowloon, China (SAR)

† Joint last authors

*Corresponding author:
Marianna Purgato
Email: marianna.purgato@univr.it
Postal address:
WHO Collaborating Centre for Research and Training in Mental Health and Service Evaluation, Ospedale Policlinico Rossi,
Piazzale LA Scuro 10,
37134, Verona, Italy
Phone number: +390458124884

Word count: 4044

Key words: randomized controlled trials, psychosocial interventions, asylum seekers, refugees, global mental health

For peer review only

Abstract

Introduction: This article describes two randomized controlled trials (RCTs) that will evaluate the effectiveness and cost-effectiveness of Self Help Plus (SH+), a group self-help intervention developed by the World Health Organization to reduce distress. In these trials SH+ is being tested as a preventative intervention to lower the incidence of mental disorders in asylum seekers and refugees with psychological distress resettled in Europe and Turkey.

Methods and analysis: Two prospective, multicentre, randomized, rater-blinded, parallel-group studies will follow participants over a period of 12 months. One trial will be conducted in Europe and one in Turkey. In each trial, 600 asylum seekers and refugees screening positive on the General Health Questionnaire [1] (≥ 3), but without a formal diagnosis of any mental disorders according to the Mini International Neuropsychiatric Interview (M.I.N.I.), will be randomly allocated to SH+ or to enhanced treatment-as-usual. The primary outcome will be a lower incidence of mental disorders at 6-month follow-up. Secondary outcomes will include the evaluation of psychological symptoms, functioning, well-being, treatment acceptability, and indicators of intervention cost-effectiveness.

Ethics and dissemination: The two trials received ethical clearance from the local Ethics Committees of the participating sites (seven sites), as well as from the WHO Ethics Committee. All participants will provide informed consent before screening and before study inclusion (a two-step procedure). The results of the trials will be disseminated in agreement with a dissemination plan that includes publication(s) in peer-reviewed journals and presentations at relevant national and international conferences and meetings.

Trials registration numbers: NCT03571347 (European trial); NCT03587896 (Turkish trial).

Strengths and limitation of this study

► Interventions for preventing mental disorders are a public health priority.
► Evaluating the effectiveness of a low-intensity group self-help intervention is particularly relevant in emergency and limited-resource settings.
► Conducting research with populations in transit is challenging, as participants might not be traceable at follow-up, and may not be interested in participating in clinical studies.
► The possibility of contamination between the experimental and control arms cannot be excluded, as randomization will occur at the individual level.

For peer review only

Introduction

Background and rationale

The number of people seeking refugee status in European and bordering countries has progressively increased in recent years, driven by the wars in Syria, Afghanistan, and Iraq, alongside conflicts and instability in Afghanistan and elsewhere[2-4]. These populations face numerous challenges before, during, and after migration[5-9]. Refugees and asylum seekers are exposed to multiple stressors in their native countries (e.g., war-related experiences, violence, destructions of their homes). They are also exposed to many stressors during migration[7,10]. After arriving in host countries, they may then face additional challenges and perceived discrimination due to language barriers, loss of family and community support, poverty, lack of access to social, educational and healthcare institutions, and uncertain asylum application procedures[11,12].

These factors place refugees and asylum seekers at considerable risk for developing common mental disorders, including post-traumatic stress disorder (PTSD), anxiety disorders and depressive disorders, and other forms of disabling psychological distress[13,14].

There is a growing body of research demonstrating the effectiveness of psychological therapies for refugees and asylum seekers[13,15]. A recently-published umbrella review of systematic reviews on this topic showed encouraging results in terms of reduction of depression, PTSD, and other anxiety symptoms[13]. However, treatments typically require extensive training and supervision, and sufficient time to be delivered in full (e.g., up to 12 weeks). Additionally, most interventions targeted the symptoms of mental disorders, without a specific focus on prevention[9,13,16-18].

Recently, the World Health Organization (WHO) has developed a novel, low-intensity five-session self-help intervention for managing stress and coping with adversity, which is designed to be delivered by non-specialist facilitators and does not require extensive training[19]. This psychosocial intervention, called Self-Help Plus (SH+), is intended to help people with and without mental disorders to cope with distress stemming from diverse types of adversity. SH+ has already been tested in small pilot studies[20,21] and a large positive RCT with South Sudanese female refugees in Uganda[20,21]. SH+ has not been tested as a preventive intervention.

In order to fill this gap, two randomized controlled trials (RCTs) were designed to test SH+ as an indicated preventive intervention with asylum seekers and refugees resettled in European countries and in Turkey. The choice of conducting two separate trials is mainly due to the type of setting, and the different traveling experiences of asylum seekers and refugees.

Aim and objectives

The overall aim of these RCTs is to evaluate the effectiveness and cost-effectiveness of SH+ as an indicated preventive intervention in asylum seekers and refugees with psychological distress resettled in six sites of five European countries (Italy, Austria, Germany, Finland, and two sites in the UK), and in Turkey, as compared with enhanced treatment as usual (ETAU). The primary objective is the reduction in the incidence of any mental disorder at 6-month follow-up. Secondary objectives are the evaluation of psychological symptoms, functioning, well-being, treatment acceptability, and economic outcomes.

Study hypotheses

For peer review only - http://bmjopen.bmj.com/site/about/guidelines.xhtml

1. SH+ will be superior to ETAU in preventing the onset of any mental disorder at 6-month follow-up.

2. Compared to the ETAU group, asylum seekers and refugees in the SH+ intervention arm will report an improvement in depression, anxiety and PTSD symptoms; psychosocial well-being; and improved levels of psychological functioning.

3. Asylum seekers and refugees in the SH+ intervention arm will incur lower health care costs compared to the ETAU group.

Methods and analyses

These are two prospective, multicentre, randomized, parallel-group studies that will follow participants over a period of 12 months. Figure 1 provides a flow-chart of the RCT's process.

Figure 1 here

Refugees and asylum seekers with psychological distress as assessed by the General Health Questionnaire (GHQ) [1], but without a mental disorder according to the Mini International Neuropsychiatric Interview (M.I.N.I.)[22] for the Diagnostic and Statistical Manual of Mental Disorders, 5th edition (DSM-5)[23] and the ICD-10 classification of mental and behavioural disorders[24], will be randomly assigned to SH+ or ETAU. Participants will be assessed at baseline before randomization, immediately post-intervention, and at 6 and 12-months of follow-up. The two RCTs will be conducted in accordance to the CONSORT statement[25,26].

Inclusion criteria:
(a) Age 18 or above;
(b) Able to speak and understand one of the languages in which SH+ has been adapted: Arabic, Dari, Urdu, or English;
(c) Asylum seeker or refugee or person under Temporary Protection;
(d) Presence of psychological distress, as shown by a score of 3 or more on the 12-item GHQ-12;
(e) Both oral and written informed consent to enter the study.

Exclusion criteria:
(a) Presence of any mental disorders according to DSM-5 and ICD-10, as shown by a positive M.I.N.I.;
(b) Acute medical conditions contraindicating study participation, based on the clinical judgment of the health care professional or trained paraprofessional who performs the screening;
(c) Clinical evidence of imminent suicide risk or suicide risk scored as "moderate or high" (or a positive suicidality behaviour disorder) by the M.I.N.I. (section Suicidality);
(d) Clinical evidence that decision-making capacity is impaired.

Asylum seekers and refugees that are excluded because of a diagnosis of a mental disorder will be advised to seek professional treatment according to a pre-defined protocol.

For peer review only - http://bmjopen.bmj.com/site/about/guidelines.xhtml

Two experts giving advice on any ethical issues related to the trials were appointed to be members of an Ethics Advisory Board (EAB). Additionally, a data Protection Officer (DPO) was appointed to supervise and report that data collection is carried out according to European and national legislations.

Characteristics of the intervention

The SH+ programme has been developed by WHO and collaborators working in the humanitarian field. SH+ consists of a pre-recorded audio course, delivered by facilitators in a group setting and complemented with an illustrated self-help book. The potential value of using a psychoeducational course to access hard-to-reach populations has been shown previously [27]. Evidence for the use of books in interventions is also promising[28]. Furthermore, research has found that guided self-help programs produce much better results than "pure" self-help, and the effects produced are similar to face-to-face psychological treatment[29]. SH+ was designed to be relevant for large segments of adversity-affected populations: it is intended to be transdiagnostic, easily adaptable to different cultures and languages, and both meaningful and safe for people with and without mental disorders.

The format of SH+ is innovative in that it seeks to ensure that key intervention components are delivered as intended without the burden of extensive facilitator training. The SH+ programme is based on acceptance and commitment therapy (ACT), a form of cognitive-behavioral therapy, with distinct features[30]. ACT is based on the concept that ongoing attempts to suppress unwanted thoughts and feelings can paradoxically make these problems worse. Instead, it emphasizes learning new ways to accommodate difficult thoughts and feelings, while guiding people to take proactive steps towards living in a way that is consistent with their values. ACT has been shown to be useful for a range of mental health issues [31] and has been used successfully in a guided self-help format[32]. SH+ includes ACT techniques that aim to help people cope with stress, respond compassionately to themselves and others, and to live in accordance with their values.

The SH+ programme has two components: a pre-recorded course and an illustrated self-help book. Pre-recorded audio material is delivered across five 2-hour sessions to groups of up to 30 people. The audio material imparts key information about stress management and guides participants through individual exercises and small group discussions. To augment the audio recordings, an illustrated self-help book reviews all essential content and concepts[19]. Written manuals help non-specialist facilitators to conduct the course using the pre-recorded audio.

Delivery of SH+

SH+ will be delivered by briefly-trained, non-specialist facilitators with a refugee or migrant background. SH+ trainers will be academics and/or mental health care professionals. Before the recruitment period, SH+ trainers received specific training on the SH+ intervention from WHO.

SH+ has been adapted into Arabic, Dari, and Urdu according to the WHO Department of Mental Health and Substance Abuse draft protocol for adapting psychological interventions for common mental health problems. An English version is also available, which has been translated into the other languages with a focus on ensuring the translated version text and images are acceptable, understandable and relevant to the population. Close attention was paid to the form of language (e.g. colloquial) and tone (e.g. warm and caring) so that it may be fully suitable for a pre-recorded audio psychological intervention. The SH+ audio and book were reviewed and revised by native speakers with diverse backgrounds.

For peer review only - http://bmjopen.bmj.com/site/about/guidelines.xhtml

Enhanced Treatment as Usual (ETAU)

Control arm participants will receive routine social support and/or care following local regulations. Additionally, they will receive baseline and follow-up assessments according to the study schedule; information about freely available health and social services, and community networks that provide support to refugees and asylum seekers.

Primary and secondary outcomes

Outcome measures and time-points are detailed in table 1. The primary outcome will be the incidence of any mental disorders measured by the M.I.N.I. at 6-month follow-up. All other measures will be secondary outcomes.

For peer review only

Concept	Screening	Baseline		Post-intervention	6-month follow-up	12-month follow-up
	Informed consent	Informed consent	RANDOMIZATION			
**Mental capacity**	Mental capacity form	Mental capacity form		Mental capacity form	Mental capacity form	Mental capacity form
**Psychological distress**	GHQ-12			GHQ-12	GHQ-12	GHQ-12
**Psychiatric diagnosis**	MINI			MINI	MINI	MINI
**Socio-Demographic and migration data**		Recruitment form				
**Symptoms of PTSD**		PCL-5		PCL-5	PCL-5	PCL-5

For peer review only - http://bmjopen.bmj.com/site/about/guidelines.xhtml

**Depressive symptoms**		PHQ-9		PHQ-9	PHQ-9	PHQ-9
**Adverse life events**		HTQ –Part A/1				
**Subjective wellbeing**		WHO-5 Wellbeing index		WHO-5 Wellbeing index	WHO-5 Wellbeing index	WHO-5 Wellbeing index
**Health-related quality of life**		EQ-5D-3L			EQ-5D-3L	EQ-5D-3L
**Self-defined psychosocial goals**		PSYCHLOPS (pre-int. version)		PSYCHLOPS (post-int. version)	PSYCHLOPS (post-int. version)	PSYCHLOPS (post-int. version)
**Post migration stress difficulties**				PMLD	PMLD	PMLD
**Psychological functioning**		WHODAS 2.0		WHODAS 2.0	WHODAS 2.0	WHODAS 2.0
**Cost-effectiveness**		CSSRI-EU			CSSRI-EU	CSSRI-EU
**Adverse events**					**Adverse events form**	

Table 1. Outcome measures and time points

Legend: General Health Questionnaire-12 items (GHQ-12); Mini International Neuropsychiatric Interview (MINI); PTSD Checklist for DSM-V (PCL-5); Patient Health Questionnaire – 9 items (PHQ-9); Harvard Trauma Questionnaire (HTQ); World Health Organization – 5 items (WHO-5) Wellbeing Index; Three-level version of EuroQol Group (EQ-5D-3L); Psychological Outcome Profiles Instrument (PSYCHLOPS); World Health Organization Disability Assessment Schedule (WHODAS); Client Socio-Demographic and Service Receipt Inventory, European Version (CSSRI-EU).

Randomization

Randomization will occur at the individual level and will be stratified by each recruiting centre. To avoid contamination, only one person per household will be randomized. Randomization will be centralized and coordinated by the WHO Collaborating Centre of the University of Verona. Eligible participants will be randomly assigned to one of the two groups with an equal probability of assignment to each group (allocation ratio 1:1). The randomization schedule will be generated using the web-based software Castor Electronic Data Capture[33]. This electronic tool employs a variable block randomization method, in order to allocate groups randomly permuted in blocks of unequal size. The site investigators will not know the block size and will not be able to access the randomization list. The randomization list will be accessible only to the data manager. In addition, the web-based software will allow random allocation only after

For peer review only - http://bmjopen.bmj.com/site/about/guidelines.xhtml

the main information on the enrolled participant is entered, upon verification of the inclusion criteria. After random allocation, the software will produce a unique identification number for each participant. In accordance with the Declaration of Helsinki[34], the participants' confidentiality will be preserved at all times and the contents of the recruitment and follow-up forms will not be disclosed to any third party.

Masking

Masking participants and facilitators about the intervention status will be impossible, due to the nature of the intervention. However, investigators evaluating primary and secondary outcomes post-intervention and at 6- and 12-month follow-up, and the statistician performing all analyses, will be masked to the participants' allocation status. Health care professionals and cultural mediators involved in the assessments will be instructed on how to perform follow-up assessments in order to preserve effective masking.

Sample size and power calculations

On the basis of data extrapolated from prevention trials, an event rate (diagnosis of any mental disorders) of around 15% at six months (primary outcome) is expected[35,36]. However, these prevention trials were conducted in unselected populations that were not exposed to migration stressors. By contrast, refugees and asylum seekers are exposed to stressors that are associated with increased rates of mental disorders[9]. On these grounds, we anticipate an incidence rate of mental disorders of 25% at six months in this population. It is hypothesized that the provision of the SH+ programme will show a clinically significant advantage by producing a between-groups absolute difference of 10%. A sample size of 500 participants per trial, achieves 80% power for a 0.05 level of significance between the two proportions of people diagnosed with mental disorders at six months. Assuming that a relevant proportion of asylum seekers and refugees might be lost at study endpoint (due to the specific characteristics of this population), the final sample size will be of 600 participants (300 in each group) for the European trial and 600 participants (300 in each group) for the Turkish trial. We anticipate that we will screen around 1800-2000 people to have 600 eligible participants.

Adverse events reporting

Serious adverse events and other adverse events reported spontaneously by the participants or observed by the research staff will be recorded on a specifically developed form. Data on the relationship with the study intervention, the action taken regarding intervention, and the outcome of the adverse event, will be collected. An event is considered a potential adverse reaction if it is an undesirable experience occurring to a participant during the study, whether or not it is considered related to the research procedure. This definition includes all aspects of mental health and psychological functioning, but also any other undesirable experiences. The EAB will review spontaneously reported serious adverse reactions (e.g., suicide attempts) within 48h, while general adverse reactions will be reviewed by the EAB in regular meetings.

Data management and statistical analysis

Statistical Analysis

General approach

The statistical analysis will be masked. All analyses will be performed using Stata/SE, Release 14.2.

Two data locks will occur during the study. The first will happen 6 months after the end of the 12-month enrolment period, when information on the primary outcome and on short-term

For peer review only - http://bmjopen.bmj.com/site/about/guidelines.xhtml

secondary outcomes will be available for all the participants. The second will happen 12 months after the end of the 12-month enrolment period. All primary and secondary analyses will be performed on an intention-to-treat (ITT) basis. The ITT population will consist of all participants randomly assigned to the competing intervention strategies, and with data on the baseline assessment available. In order to check the robustness of results, the primary outcome will be additionally analyzed using a per protocol (PP) approach, that will include only SH+ participants who attended at least three sessions. The analysis of the PP population will be used for confirmatory purposes only. If less than 5% of participants do not receive the allocated intervention according to the study protocol, the PP analysis will not be performed. All analyses reaching statistical significance will be replicated for each recruiting centre and for each target language separately.

Analysis of the primary outcome

The proportion of participants with a diagnosis of any mental disorders at 6-months follow-up will be compared between the two groups using a chi-square test (primary analysis). A multivariate analysis (secondary analysis) will be performed through a Poisson regression model, with robust error variance [34] , to estimate relative risks directly and to explore the potential confounding effect of prognostic factors, and the interactions with treatment.

Analysis of the secondary outcomes

The proportion of participants with a diagnosis of any mental disorder post-intervention and at 12-months of follow-up will be compared between the two groups using a chi-square test. A multivariate analysis will be performed using a Poisson regression model, with a robust error variance, to estimate relative risks directly and to explore the potential confounding effect of prognostic factors, and the interactions with treatment[37].

Analysis of GHQ-12, WHODAS 2.0, PHQ-9, WHO-5 Wellbeing index, PSYCHLOPS, PCL-5

For each questionnaire, in case of missing items, the Corrected Item Mean Substitution method[38], will be used (i.e. the item mean across participants weighted by the subject's mean of completed items), using information from subjects belonging to the same treatment arm for the same follow-up time. The hypothesis that the experimental intervention has no effect on GHQ-12, WHODAS 2.0, PHQ-9, WHO-5 Wellbeing index, PSYCHLOPS, PCL-5 scores will be tested by performing seemingly unrelated regression[39], for each time point, controlling for baseline values. In case of joint statistical significance of the coefficients related to treatment status, the effect of treatment on each score will be evaluated through a mixed analysis of covariance (ANCOVA), controlling for the value at baseline. After the last data lock, ANCOVA with robust standard errors will be performed for each scale, using the values at post-intervention, and at 6- and 12-months follow-up, controlling for the value at baseline. In case of joint statistical significance of the coefficients related to treatment status, the analysis will be repeated for each outcome/time combination. Such approaches (assessing the global significance of all time locks for each scale, without imputing missing values) will allow to have a robustness check to results obtained after each time point. As a further sensitivity check, multivariate analyses will be performed for each scale to take confounding factors into account, again including the baseline value as a covariate.

The proportion of participants leaving the study early will be compared between the two groups using a chi-square test (or the Fisher exact test, where appropriate). A multivariate analysis will be performed using a Poisson regression model, with a robust error variance, to estimate

For peer review only - http://bmjopen.bmj.com/site/about/guidelines.xhtml

relative risks directly and to explore the potential confounding effect of prognostic factors, and the interactions with treatment[37].

Cost-effectiveness analysis

Incremental cost-effectiveness analyses (ICEA) will be conducted from the health and social care system perspective and from the societal perspective by means of the net-benefit approach[40]. For the five European countries the ICEA will be conducted from the perspective of the UK health care system and for Turkey from the perspective of the Turkish health care system. Direct costs including formal medical and psychiatric health care, psychosocial care, legal services, and informal services will be used for ICEA from the health and social care system (payer) perspective. Total costs will be used for ICEA from the societal perspective. Comprehensive use of annual health and social care resources will be assessed by means of completing the adapted CSSRI-EU. Information on the unit-costs of the used services for the European countries will be gained by the cost compilation for the UK health and social care system[41]. For Turkey, price lists and other sources will be used to identify unit costs. Costs of informal services will be estimated for both studies on the basis of average salaries per hour. Total direct costs of service use will be calculated by multiplying service units with unit costs. Annual total costs of mental illness will be estimated on the basis of two retrospective assessments of six month service use at six and twelve month follow-up. Productivity losses will be estimated for participants with work permissions by means of the human capital approach[42] on the basis of group-specific average salaries. Costs for the European countries will be calculated in 2017 UK £. Costs for Turkey will be calculated in Turkish Lira (TL). Total costs of health and social care service use will be investigated with regard to the cost driving effects of asylum seekers and refugees characteristics and the impact of different levels of coverage, different macroeconomic, social and health care indicators by estimating regression based cost functions[43]. Incremental NMB will be computed by regressing the NMB on study group[44]. Results of ICEA for the European countries will be interpreted according to UK MWTP thresholds recommended by the NICE[45]. Results for Turkey will be interpreted according to the three times GDP per capita threshold [46] and alternatively by country specific thresholds provided by Woods et al.[47]. For international comparison all cost data and results of ICEA will be converted into € and US$ adjusted for power purchasing parities (PPP).

Patient and Public involvement

Participants have not been involved in the design of the study. However, SH+ facilitators delivering the intervention will be refugees or community members with a refugee or migrant background or with the same/similar culture of participants. They will have some prior experience in health or social or community work or volunteering. The choice of asylum seekers' and refugees' country of origin was made in accordance with a situational analysis on size of refugee populations within European countries and Turkey, as well as feasibility issues.

Ethics and dissemination

It is essential to conduct this research because rigorous evidence needs to be collected on the effectiveness of the SH+ intervention, which has been specifically developed for vulnerable populations. Additionally, it will provide valuable information about optimal adaptation strategies and aspects to consider when scaling up the intervention in diverse contexts.

The trials will be conducted according to globally accepted standards of good clinical practice (as defined in the ICH E6 Guideline for Good Clinical Practice, 1 May 1996), in agreement with the Declaration of Helsinki and in keeping with local regulations.

Dissemination includes a publication plan agreed by all members of the Consortium that regulates academic articles and conference presentations. On the basis of positive results from

For peer review only - http://bmjopen.bmj.com/site/about/guidelines.xhtml

research trials of SH+, the SH+ package will be disseminated by WHO in multiple languages.

DISCUSSION

This article presents the main characteristics of two RCTs, which will assess the effectiveness and cost-effectiveness of a low-intensity self help intervention to prevent mental disorders among distressed refugees and asylum seekers. The concept of low-intensity implies that fewer resources are used than in more complex, conventional psychosocial treatments. SH+ may be delivered by facilitators without formal mental health professional training, employs low-cost technologies, such as audio recordings and an illustrated book, has only 5 sessions, and may be delivered to groups of up to 30 participants. The group setting has the advantage of being scalable and optimizing resource use, but it has limited scope for in-depth work with individuals, and participants exposed to traumatic experiences might be concerned about interacting with others in a group space.

If positive results from these trials are achieved, WHO may adopt and disseminate SH+ as a preventive intervention in multiple languages.

A number of characteristics of the design of these two trials may hamper or facilitate their implementation. First, the study was designed as two trials with an almost identical design, with one conducted in European countries and one in Turkey. We reasoned that even though the population that will be recruited in Turkey shares important characteristics with the population in European countries, in terms of pre-migration, migration and post migration stressors[6,10,48], the resettlement context is likely to be different. This may be relevant for a number of aspects, including the definition of the control group, which receives treatment as usual enhanced by referral mechanisms to social organizations and health care facilities. While these mechanisms may show some similarities across different European countries, the same may not apply for Turkey. The resettlement context may additionally be relevant for the capacity of research staff to follow participants, as this aspect may be substantially affected by the mobility of the population under study.

Second, a focus on prevention, rather than treatment, is challenging. In practical terms, it means recruiting participants showing some psychological distress but not a mental disorder. This may represent an obstacle to recruitment, as at study entry the administration of the MINI, which is of paramount relevance to exclude those with a disorder, requires time and training. Additionally, since participants who screen positive cannot be included, we anticipate the need to screen hundreds of potential participants to reach the target sample size. Another aspect related to the recruitment of participants without a disorder is that those eligible for inclusion will clearly be less distressed as compared with those suffering from a mental disorder. Therefore, eligible participants may be less likely to agree to be included, as they may feel that dealing with their psychological distress may not be as relevant as dealing with other social or health aspects. Participants with a mental disorder, by contrast, may recognise that dealing with psychological problems is a high priority and a pre-requisite for optimal social functioning. This may be another obstacle for effective recruitment.

Despite these potential challenges, establishing the effectiveness of preventive psychosocial interventions is a public health priority, as it may help large population groups over short periods of time. Preventive psychosocial interventions must be feasible, sustainable, and cost-effective. SH+, which is based on self-help approaches, may have particular advantages for populations with limited access to social and health care services, and with high levels of need.

For peer review only - http://bmjopen.bmj.com/site/about/guidelines.xhtml

CONCLUSION

This project aims to generate a strong evidence-base for preventative aspects of SH+, and to create a scientific framework for adapting and equipping social and health care systems in countries inside and outside Europe with such a low intensity intervention. The delivery format of SH+ is innovative, as it requires a short training of non-professional facilitators, and reduced supervision, and fidelity is assured by the pre-recorded audio and the book. Moreover, the intervention has been culturally adapted according to a WHO protocol and cultural adaptation will be extended to other population groups after publication of the SH+ programme.

Authors' contributions

The study was conceived and designed by Mark van Ommeren and Corrado Barbui. Marianna Purgato and Corrado Barbui drafted the manuscript. Ceren Acarturk, Teresa Au, Ken Carswell, Michela Nosè, Giovanni Ostuzzi, Federico Tedeschi, Giulia Turrini, and Mark van Ommeren contributed to the study design and commenting/editing the manuscript drafts.
All authors contributed revising and commenting the manuscript drafts. The authors alone are responsible for the views expressed in this article and they do not necessarily represent the views, decisions or policies of the institutions with which they are affiliated.

Ethics Advisory Board members: Anna Chiumento, Stefan Weinmann

Funding statement

This work is supported by the European Commission, grant agreement n. 779255 "RE-DEFINE: Refugee Emergency: DEFining and Implementing Novel Evidence-based psychosocial interventions".

Competing interests statement

None.

For peer review only

For peer review only - http://bmjopen.bmj.com/site/about/guidelines.xhtml

For peer review only

References

1. Goldberg DP, Blackwell B. Psychiatric illness in general practice. A detailed study using a new method of case identification. *Br Med J* 1970;1:439-443.

2. UNHCR. Global trends. Forced displacement in 2017. Available from https://www.unhcr.org/globaltrends2017/ Accessed March 6, 2019.

3. UNHCR. Global trends. Forced displacement in 2015. Availeble from https://www.unhcr.org/statistics/unhcrstats/576408cd7/unhcr-global-trends-2015.html Accessed March 6, 2019.

4. UNHCR. Global trends. Forced displacement in 2016. Available from https://www.unhcr.org/statistics/unhcrstats/5943e8a34/global-trends-forced-displacement-2016.html Accessed March 6, 2019.

5. Wells R, Steel Z, Abo-Hilal M, Hassan AH, Lawsin C. Psychosocial concerns reported by Syrian refugees living in Jordan: systematic review of unpublished needs assessments. *Br J Psychiatry* 2016;209:99-106.

For peer review only - http://bmjopen.bmj.com/site/about/guidelines.xhtml

6. Kirmayer LJ, Narasiah L, Munoz M, *et al*. Common mental health problems in immigrants and refugees: general approach in primary care. *CMAJ* 2011;183:E959-E967.

7. Miller KE, Rasmussen A. The mental health of civilians displaced by armed conflict: an ecological model of refugee distress. *Epidemiol Psychiatr Sci* 2016:1-10.

8. World Health Organization Regional Office for Europe. Mental health promotion and mental health care in refugees and migrants. Technical guidance. Copenhagen:WHO;2018.

9. Priebe S, D G, R E-N. Public health aspects of mental health among migrants and refugees: a review of the evidence on mental health care for refugees, asylum seekers and irregular migrants in the WHO European Region (2016). In:2016.

10. Miller KE, Rasmussen A. War exposure, daily stressors, and mental health in conflict and post-conflict settings: bridging the divide between trauma-focused and psychosocial frameworks. *Soc Sci Med* 2010;70:7-16.

11. Alemi Q, Stempel C. Discrimination and distress among Afghan refugees in northern California: The moderating role of pre- and post-migration factors. *PLoS One* 2018;13:e0196822.

12. Purgato M, Tol WA, Bass JK. An ecological model for refugee mental health: implications for research. *Epidemiol Psychiatr Sci* 2016:1-3.

13. Turrini G, Purgato M, Ballette F, Nose M, Ostuzzi G, Barbui C. Common mental disorders in asylum seekers and refugees: umbrella review of prevalence and intervention studies. *Int J Ment Health Syst* 2017;11:51.

14. Zhong BL, Liu TB, Chan SSM, et al. Common mental health problems in rural-to-urban migrant workers in Shenzhen, China: prevalence and risk factors. *Epidemiol Psychiatr Sci* 2018;27:256-265.

15. Nose M, Ballette F, Bighelli I, et al. Psychosocial interventions for post-traumatic stress disorder in refugees and asylum seekers resettled in high-income countries: Systematic review and meta-analysis. *PLoS One.* 2017;12(2):e0171030.

16. Giacco D, Laxhman N, Priebe S. Prevalence of and risk factors for mental disorders in refugees. *Semin Cell Dev Biol* 2017.

17. Giacco D, Priebe S. Mental health care for adult refugees in high-income countries. *Epidemiol Psychiatr Sci* 2017:1-8.

18. Turrini G, Purgato M, Acarturk C, *et al.* Efficacy and acceptability of psychosocial interventions in asylum seekers and refugees: systematic review and meta-analysis. *Epidemiol Psychiatr Sci* 2019;11:1-13.

19. Epping-Jordan JE, Harris R, Brown FL, et al. Self-Help Plus (SH+): a new WHO stress management package. *World Psychiatry* 2016;15:295-296.

For peer review only - http://bmjopen.bmj.com/site/about/guidelines.xhtml

20. Tol WA, Augustinavicius J, Carswell K, et al. Translation, adaptation, and pilot of a guided self-help intervention to reduce psychological distress in South Sudanese refugees in Uganda. *Glob Ment Health (Camb)* 2018;5:e25.

21. Tol WA, Augustinavicius J, Carswell K, et al. Feasibility of a guided self-help intervention to reduce psychological distress in South Sudanese refugee women in Uganda. *World Psychiatry* 2018;17:234-235.

22. Sheehan DV, Lecrubier Y, Sheehan KH, *et al*. The Mini-International Neuropsychiatric Interview (M.I.N.I.): the development and validation of a structured diagnostic psychiatric interview for DSM-IV and ICD-10. *J Clin Psychiatry* 1998;59:22-33.

23. American Psychiatric Association. The Diagnostic and Statistical Manual of Mental Disorders (5th ed.; DSM–5). Washington DC: American Psychiatric Association; 2013.

24. World Health Organization, Geneva. The ICD-10 Classification of mental and behavioural disorders: clinical descriptions and diagnostic guidelines. 1992.

25. Begg C, Cho M, Eastwood S, et al. Improving the quality of reporting of randomized controlled trials. The CONSORT statement. *JAMA.* 1996;276:637-639.

26. Schulz KF, Altman DG, Moher D. CONSORT 2010 statement: Updated guidelines for reporting parallel group randomised trials. *J Pharmacol Pharmacother* 2010;1:100-107.

27. Cuijpers P, Munoz RF, Clarke GN, *et al*. Psychoeducational treatment and prevention of depression: the "Coping with Depression" course thirty years later. *Clin Psychol Rev* 2009;29:449-458.

28. den Boer PC, Wiersma D, Van den Bosch RJ. Why is self-help neglected in the treatment of emotional disorders? A meta-analysis. *Psychol Med* 2004;34:959-971.

29. World Health Organization, Geneva. Intervention Guide for Mental, Neurological and Substance Use Disorders in Non-Specialized Health Settings: Mental Health Gap Action Programme (mhGAP). WHO:Geneva;2010.

30. Hayes SC, Levin ME, Plumb-Vilardaga J, *et al*. Acceptance and commitment therapy and contextual behavioral science: examining the progress of a distinctive model of behavioral and cognitive therapy. *Behav Ther* 2013;44:180-198.

31. Tjak JG, Davis ML, Morina N, *et al*. A meta-analysis of the efficacy of acceptance and commitment therapy for clinically relevant mental and physical health problems. *Psychother Psychosom* 2015;84:30-36.

32. Fledderus M, Bohlmeijer ET, Pieterse ME, Schreurs KM. Acceptance and commitment therapy as guided self-help for psychological distress and positive mental health: a randomized controlled trial. *Psychol Med* 2012;42:485-495.

33. Castor Electronic Data Capture, Ciwit BV, Amsterdam, The Netherlands, 2018.

For peer review only - http://bmjopen.bmj.com/site/about/guidelines.xhtml

34. World Medical Association. World Medical Association Declaration of Helsinki: ethical principles for medical research involving human subjects. *JAMA* 2013;310:2191-2194.

35. Cuijpers P, van SA, Smit F, *et al*. Preventing the onset of depressive disorders: a meta-analytic review of psychological interventions. *Am J Psychiatry* 2008;165:1272-1280.

36. van Zoonen K, Buntrock C, Ebert DD, *et al*. Preventing the onset of major depressive disorder: a meta-analytic review of psychological interventions. *Int J Epidemiol* 2014;43:318-329.

37. Zou G. A modified poisson regression approach to prospective studies with binary data. *Am J Epidemiol* 2004;159:702-706.

38. Huisman M. Item nonresponse: occurrence, causes, and imputation of missing answers to test items. Leiden: DSWO Press; 1999.

39. Zellner A. An Efficient Method of Estimating Seemingly Unrelated Regressions and Tests for Aggregation Bias. *J Am Stat Assoc* 1962;57:348-368.

40. Glick H. Economic evaluation in clinical trials (Reprinted.). In: Press OU, ed. Handbooks in Health Economic Evaluation Series.2010.

41. Curtis L, Burns A. Unit Costs of Health and Social Care 2017. 2017.

42. Brouwer WB, Koopmanschap MA. How to calculate indirect costs in economic evaluations. *Pharmacoeconomics* 1998;13:563-569.

43. Knapp M. Making music out of noise--the cost function approach to evaluation. *Br J Psychiatry Suppl* 1998:7-11.

44. Hoch JS, Briggs AH, Willan R. Something old, something new, something borrowed, something blue: a framework for the marriage of health econometrics and cost-effectiveness analysis. *Health Econ* 2002;11:415-430.

45. Appleby J. Crossing the line: NICE's value for money threshold. *BMJ* 2016;352:i1336.

46. Robinson LA, Hammitt JK, Chang AY, *et al*. Understanding and improving the one and three times GDP per capita cost-effectiveness thresholds. *Health Policy Plan* 2017;32:141-145.

47. Woods B, Revill, P, Sculpher, M, *et al*. Country-Level Cost-Effectiveness Thresholds: Initial Estimates and the Need for Further Research. *Value in Health* 2016;19:929-935.

48. Sijbrandij M, Acarturk C, Bird M, *et al*. Strengthening mental health care systems for Syrian refugees in Europe and the Middle East: integrating scalable psychological interventions in eight countries. *Eur J Psychotraumatol* 2017;8:1388102.

For peer review only - http://bmjopen.bmj.com/site/about/guidelines.xhtml

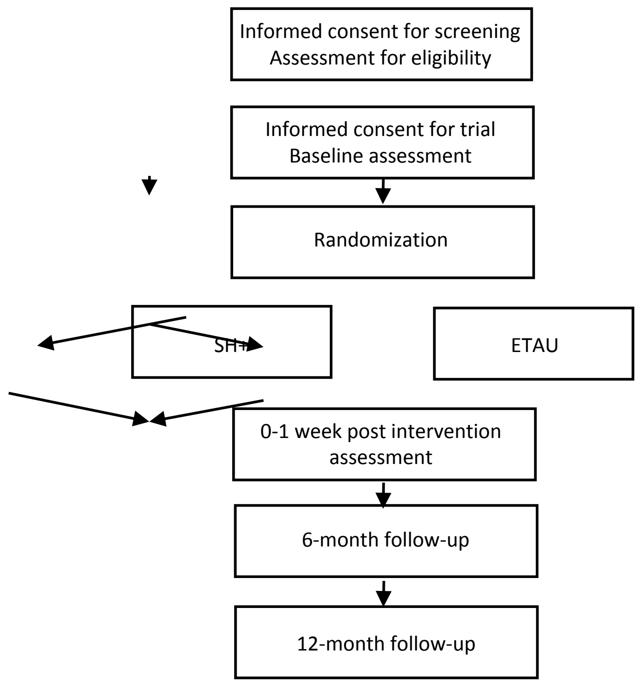

Figure 1. RCT Flow diagram

BMJ Open

Effectiveness and cost-effectiveness of Self Help Plus (SH+) for preventing mental disorders in refugees and asylum seekers in Europe and Turkey: study protocols for two randomized controlled trials

Journal:	*BMJ Open*
Manuscript ID	bmjopen-2019-030259.R1
Article Type:	Protocol
Date Submitted by the Author:	03-Apr-2019

Complete List of Authors:	Purgato, Marianna; University of Verona, Department of Neurosciences, Biomedicine and Movement Sciences; Cochrane Global Mental Health<br>Carswell, Kenneth; World Health Organization, Department of Mental Health & Substance Abuse<br>Acarturk, Ceren; Istanbul Sehir Universitesi,<br>Au, Teresa; World Health Organization, Department of Mental Health & Substance Abuse<br>Akbai, Sena; Istanbul Sehir Universitesi, Department of Psychology<br>Anttila, Minna; University of Turku, Department of Nursing Science<br>Baumgartner, Josef; Medical University of Vienna, Department of Psychiatry and Psychotherapy<br>Bailey, Della; University of York, Health Sciences<br>Biondi, Massimo; University of Rome La Sapienza, Department of Human Neurosciences<br>Bird, Martha; Danish Red Cross, IFRC Reference Centre for Psychosocial Support<br>Churchill, R; University of York,<br>Eskici, Sevde; Istanbul Sehir Universitesi, Department of Psychology<br>Hansen, Louise; Danish Red Cross, IFRC Reference Centre for Psychosocial Support<br>Heron, Paul; University of York, Department of Health Sciences<br>Ilkkursun, Zeynep; Istanbul Sehir Universitesi, Department of Psychology<br>Kilian, Reinhold; Ulm University, Department of Psychiatry II<br>Koesters, Markus; Ulm University, Department of Psychiatry II; Cochrane Global Mental Health<br>Lantta, Tella; Turun Yliopisto, Department of Nursing Science<br>Nosè, Michela; University of Verona, Department of Neurosciences, Biomedicine and Movement Sciences; Cochrane Global Mental Health<br>Ostuzzi, Giovanni; University of Verona, Department of Neurosciences, Biomedicine and Movement Sciences; Cochrane Global Mental Health<br>Papola, Davide; University of Verona, Department of Neurosciences, Biomedicine and Movement Sciences; Cochrane Global Mental Health<br>Popa, Mariana; University of Liverpool, Institute of Life and Human Sciences<br>Sijbrandij, Marit; Vrije Universiteit Amsterdam,<br>Tarsitani, Lorenzo; University of Rome La Sapienza, Department of Human Neurosciences

	Tedeschi, Federico; University of Verona, Department of Neurosciences, Biomedicine and Movement Sciences<br>Turrini, Giulia; University of Verona, Department of Neurosciences, Biomedicine and Movement Sciences; Cochrane Global Mental Health<br>Uygun, Ersin; Istanbul Bilgi Universitesi, Trauma and Disaster Mental Health<br>Välimäki, Maritta; University of Turku, Department of Nursing Science; Hong Kong Polytechnic University, School of Nursing<br>Wancata, Johannes; Medical University of Vienna, Department of Psychiatry and Psychotherapy, Division of Social Psychiatry<br>White, Ross; University of Liverpool, Institute of Life and Human Sciences<br>Zanini, Elisa; University of Verona, Department of Neurosciences, Biomedicine and Movement Sciences<br>Cuijpers, Pim; VU University Amsterdam, Department of Clinical Psychology<br>Barbui, Corrado; University of Verona, Department of Neurosciences, Biomedicine and Movement Sciences; Cochrane Global Mental Health<br>Van Ommeren, Mark; World Health Organization, Dep Mental Health and Substance Dependence

**Primary Subject Heading**:	Mental health
Secondary Subject Heading:	Public health, Global health, Evidence based practice
Keywords:	randomized controlled trials, psychosocial interventions, asylum seekers, refugees, global mental health

SCHOLARONE™
Manuscripts

Effectiveness and cost-effectiveness of Self Help Plus (SH+) for preventing mental disorders in refugees and asylum seekers in Europe and Turkey: study protocols for two randomized controlled trials

Marianna Purgato[1,2]*, Kenneth Carswell[3], Ceren Acarturk[4], Teresa Au[3], Sena Akbai[4], Minna Anttila[5], Josef Baumgartner[6], Della Bailey[7], Massimo Biondi[8], Martha Bird[9], Rachel Churchill[10], Sevde Eskici[4], Louise Juul Hansen[9], Paul Heron[7], Zeynep Ilkkursun[4], Reinhold Kilian[11], Markus Koesters[2,11], Tella Lantta[5], Michela Nosè[1,2], Giovanni Ostuzzi[1,2], Davide Papola[1,2], Mariana Popa[12], Marit Sijbrandij[13], Lorenzo Tarsitani[8], Federico Tedeschi[1], Giulia Turrini[1,2], Ersin Uygun[14], Maritta Valimaki[5,15], Johannes Wancata[6], Ross White[12], Elisa Zanini[1], Pim Cuijpers[13], Corrado Barbui[1,2†], Mark van Ommeren[3†]

1. WHO Collaborating Centre for Research and Training in Mental Health and Service Evaluation, Department of Neuroscience, Biomedicine and Movement Sciences, Section of Psychiatry, University of Verona, Verona, Italy.

2. Cochrane Global Mental Health, University of Verona, Verona, Italy.

3. Department of Mental Health & Substance Abuse, World Health Organization, Geneva, Switzerland

4. Department of Psychology, Istanbul Sehir University, Istanbul, Turkey

5. University of Turku, Faculty of Medicine, Department of Nursing Science, Finland

6. Clinical Division of Social Psychiatry, Department of Psychiatry and Psychotherapy, Medical University of Vienna, Vienna, Austria

7. Department of Health Sciences, University of York, York, UK

8. Department of Human Neurosciences, Sapienza University of Rome, Italy

9. Danish Red Cross, IFRC Reference Centre for Psychosocial Support, Copenhagen, Denmark

10. Centre for Reviews and Dissemination, University of York, York, UK

11. Department of Psychiatry II, Ulm University, Ulm, Germany

For peer review only

12. Institute of Life and Human Sciences, University of Liverpool, UK

13. Department of Clinical, Neuro- and Developmental Psychology, Amsterdam Public Health research institute, Vrije Universiteit, Amsterdam, The Netherlands

14. Trauma and Disaster Mental Health, Istanbul Bilgi University, Istanbul, Turkey

15. Hong Kong Polytechnic University, School of Nursing, Hong Hom, Kowloon, China (SAR)

† Joint last authors

*Corresponding author:
Marianna Purgato
Email: marianna.purgato@univr.it
Postal address:
WHO Collaborating Centre for Research and Training in Mental Health and Service Evaluation, Ospedale Policlinico Rossi,
Piazzale LA Scuro 10,
37134, Verona, Italy
Phone number: +390458124884

Word count: 4044

Key words: randomized controlled trials, psychosocial interventions, asylum seekers, refugees, global mental health

For peer review only

Abstract

Introduction: This article describes two randomized controlled trials (RCTs) that will evaluate the effectiveness and cost-effectiveness of Self Help Plus (SH+), a group self-help intervention developed by the World Health Organization to reduce distress. In these trials SH+ is being tested as a preventative intervention to lower the incidence of mental disorders in asylum seekers and refugees with psychological distress resettled in Europe and Turkey.

Methods and analysis: Two prospective, multicentre, randomized, rater-blinded, parallel-group studies will follow participants over a period of 12 months. One trial will be conducted in Europe and one in Turkey. In each trial, 600 asylum seekers and refugees screening positive on the General Health Questionnaire (≥ 3), but without a formal diagnosis of any mental disorders according to the Mini International Neuropsychiatric Interview (M.I.N.I.), will be randomly allocated to SH+ or to enhanced treatment-as-usual. The primary outcome will be a lower incidence of mental disorders at 6-month follow-up. Secondary outcomes will include the evaluation of psychological symptoms, functioning, well-being, treatment acceptability, and indicators of intervention cost-effectiveness.

Ethics and dissemination: The two trials received ethical clearance from the local Ethics Committees of the participating sites (seven sites), as well as from the WHO Ethics Committee. All participants will provide informed consent before screening and before study inclusion (a two-step procedure). The results of the trials will be disseminated in agreement with a dissemination plan that includes publication(s) in peer-reviewed journals and presentations at relevant national and international conferences and meetings.

Trials registration numbers: NCT03571347 (European trial); NCT03587896 (Turkish trial). Version 4.0, March 2019 (European and Turkish trials)

Strengths and limitation of this study

► Interventions for preventing mental disorders are a public health priority.
► Evaluating the effectiveness of a low-intensity group self-help intervention is particularly relevant in emergency and limited-resource settings.
► Conducting research with populations in transit is challenging, as participants might not be traceable at follow-up, and may not be interested in participating in clinical studies.
► The possibility of contamination between the experimental and control arms cannot be excluded, as randomization will occur at the individual level.

For peer review only - http://bmjopen.bmj.com/site/about/guidelines.xhtml

Introduction

Background and rationale

The number of people seeking refugee status in European and bordering countries has progressively increased in recent years, driven by the wars in Syria, Afghanistan, and Iraq, alongside conflicts and instability in Afghanistan and elsewhere[1-3]. These populations face numerous challenges before, during, and after migration[4-8]. Refugees and asylum seekers are exposed to multiple stressors in their native countries (e.g., war-related experiences, violence, destructions of their homes). They are also exposed to many stressors during migration[6,9]. After arriving in host countries, they may then face additional challenges and perceived discrimination due to language barriers, loss of family and community support, poverty, lack of access to social, educational and healthcare institutions, and uncertain asylum application procedures[10,11].

These factors place refugees and asylum seekers at considerable risk for developing common mental disorders, including post-traumatic stress disorder (PTSD), anxiety disorders and depressive disorders, and other forms of disabling psychological distress[12,13].

There is a growing body of research demonstrating the effectiveness of psychological therapies for refugees and asylum seekers[12,14]. A recently-published umbrella review of systematic reviews on this topic showed encouraging results in terms of reduction of depression, PTSD, and other anxiety symptoms[12]. However, treatments typically require extensive training and supervision, and sufficient time to be delivered in full (e.g., up to 12 weeks). Additionally, most interventions targeted the symptoms of mental disorders, without a specific focus on prevention[8,12,15-17].

Recently, the World Health Organization (WHO) has developed a novel, low-intensity five-session self-help intervention for managing stress and coping with adversity, which is designed to be delivered by non-specialist facilitators and does not require extensive training[18]. This psychosocial intervention, called Self-Help Plus (SH+), is intended to help people with and without mental disorders to cope with distress stemming from diverse types of adversity. SH+ has already been tested in small pilot studies[19,20] and a large positive RCT with South Sudanese female refugees in Uganda[19,20]. SH+ has not been tested as a preventive intervention.

In order to fill this gap, two randomized controlled trials (RCTs) were designed to test SH+ as an indicated preventive intervention with asylum seekers and refugees resettled in European countries and in Turkey. The choice of conducting two separate trials is mainly due to the type of setting, and the different traveling experiences of asylum seekers and refugees.

Aim and objectives

The overall aim of these RCTs is to evaluate the effectiveness and cost-effectiveness of SH+ as an indicated preventive intervention in asylum seekers and refugees with psychological distress resettled in six sites of five European countries (Italy, Austria, Germany, Finland, and two sites in the UK), and in Turkey, as compared with enhanced treatment as usual (ETAU). The primary objective is the reduction in the incidence of any mental disorder at 6-month follow-up. Secondary objectives are the evaluation of psychological symptoms, functioning, well-being, treatment acceptability, and economic outcomes.

Study hypotheses

For peer review only - http://bmjopen.bmj.com/site/about/guidelines.xhtml

1. SH+ will be superior to ETAU in preventing the onset of any mental disorder at 6-month follow-up.

2. Compared to the ETAU group, asylum seekers and refugees in the SH+ intervention arm will report an improvement in depression, anxiety and PTSD symptoms; psychosocial well-being; and improved levels of psychological functioning.

3. Asylum seekers and refugees in the SH+ intervention arm will incur lower health care costs compared to the ETAU group.

Methods and analyses

These are two prospective, multicentre, randomized, parallel-group studies that will follow participants over a period of 12 months. Figure 1 provides a flow-chart of the RCT's process.

Figure 1 here

Refugees and asylum seekers with psychological distress as assessed by the General Health Questionnaire (GHQ) [21], but without a mental disorder according to the Mini International Neuropsychiatric Interview (M.I.N.I.)[22] for the Diagnostic and Statistical Manual of Mental Disorders, 5th edition (DSM-5)[23] and the ICD-10 classification of mental and behavioural disorders[24], will be randomly assigned to SH+ or ETAU. Participants will be assessed at baseline before randomization, immediately post-intervention, and at 6 and 12-months of follow-up. The two RCTs will be conducted in accordance to the CONSORT statement[25,26].

Inclusion criteria:
(a) Age 18 or above;
(b) Able to speak and understand one of the languages in which SH+ has been adapted: Arabic, Dari, Urdu, or English;
(c) Asylum seeker or refugee or person under Temporary Protection;
(d) Presence of psychological distress, as shown by a score of 3 or more on the 12-item GHQ-12;
(e) Both oral and written informed consent to enter the study.

Exclusion criteria:
(a) Presence of any mental disorders according to DSM-5 and ICD-10, as shown by a positive M.I.N.I.;
(b) Acute medical conditions contraindicating study participation, based on the clinical judgment of the health care professional or trained paraprofessional who performs the screening;
(c) Clinical evidence of imminent suicide risk or suicide risk scored as "moderate or high" (or a positive suicidality behaviour disorder) by the M.I.N.I. (section Suicidality);
(d) Clinical evidence that decision-making capacity is impaired.

Asylum seekers and refugees that are excluded because of a diagnosis of a mental disorder will be advised to seek professional treatment according to a pre-defined protocol.

For peer review only - http://bmjopen.bmj.com/site/about/guidelines.xhtml

Two experts giving advice on any ethical issues related to the trials were appointed to be members of an Ethics Advisory Board (EAB). Additionally, a data Protection Officer (DPO) was appointed to supervise and report that data collection is carried out according to European and national legislations.

Characteristics of the intervention

The SH+ programme has been developed by WHO and collaborators working in the humanitarian field. SH+ consists of a pre-recorded audio course, delivered by facilitators in a group setting and complemented with an illustrated self-help book. The potential value of using a psychoeducational course to access hard-to-reach populations has been shown previously [27]. Evidence for the use of books in interventions is also promising[28]. Furthermore, research has found that guided self-help programs produce much better results than “pure” self-help, and the effects produced are similar to face-to-face psychological treatment[29]. SH+ was designed to be relevant for large segments of adversity-affected populations: it is intended to be transdiagnostic, easily adaptable to different cultures and languages, and both meaningful and safe for people with and without mental disorders.

The format of SH+ is innovative in that it seeks to ensure that key intervention components are delivered as intended without the burden of extensive facilitator training. The SH+ programme is based on acceptance and commitment therapy (ACT), a form of cognitive-behavioral therapy, with distinct features[30]. ACT is based on the concept that ongoing attempts to suppress unwanted thoughts and feelings can paradoxically make these problems worse. Instead, it emphasizes learning new ways to accommodate difficult thoughts and feelings, while guiding people to take proactive steps towards living in a way that is consistent with their values. ACT has been shown to be useful for a range of mental health issues [31] and has been used successfully in a guided self-help format[32]. SH+ includes ACT techniques that aim to help people cope with stress, respond compassionately to themselves and others, and to live in accordance with their values.

The SH+ programme has two components: a pre-recorded course and an illustrated self-help book. Pre-recorded audio material is delivered across five 2-hour sessions to groups of up to 30 people. The audio material imparts key information about stress management and guides participants through individual exercises and small group discussions. To augment the audio recordings, an illustrated self-help book reviews all essential content and concepts[18]. Written manuals help non-specialist facilitators to conduct the course using the pre-recorded audio.

Delivery of SH+

SH+ will be delivered by briefly-trained, non-specialist facilitators with a refugee or migrant background. SH+ trainers will be academics and/or mental health care professionals. Before the recruitment period, SH+ trainers received specific training on the SH+ intervention from WHO.

SH+ has been adapted into Arabic, Dari, and Urdu according to the WHO Department of Mental Health and Substance Abuse draft protocol for adapting psychological interventions for common mental health problems. An English version is also available, which has been translated into the other languages with a focus on ensuring the translated version text and images are acceptable, understandable and relevant to the population. Close attention was paid to the form of language (e.g. colloquial) and tone (e.g. warm and caring) so that it may be fully suitable for a pre-recorded audio psychological intervention. The SH+ audio and book were reviewed and revised by native speakers with diverse backgrounds.

For peer review only - http://bmjopen.bmj.com/site/about/guidelines.xhtml

Enhanced Treatment as Usual (ETAU)

Control arm participants will receive routine social support and/or care following local regulations. Additionally, they will receive baseline and follow-up assessments according to the study schedule; information about freely available health and social services, and community networks that provide support to refugees and asylum seekers.

Primary and secondary outcomes

Outcome measures and time-points are detailed in table 1. The primary outcome will be the incidence of any mental disorders measured by the M.I.N.I. at 6-month follow-up. All other measures will be secondary outcomes.

For peer review only

Concept	Screening	Baseline		Post-intervention	6-month follow-up	12-month follow-up
	Informed consent	Informed consent	RANDOMIZATION			
**Mental capacity**	Mental capacity form	Mental capacity form		Mental capacity form	Mental capacity form	Mental capacity form
**Psychological distress**	GHQ-12			GHQ-12	GHQ-12	GHQ-12
**Psychiatric diagnosis**	MINI			MINI	MINI	MINI
**Socio-Demographic and migration data**		Recruitment form				
**Symptoms of PTSD**		PCL-5		PCL-5	PCL-5	PCL-5

For peer review only - http://bmjopen.bmj.com/site/about/guidelines.xhtml

**Depressive symptoms**		PHQ-9		PHQ-9	PHQ-9	PHQ-9
**Adverse life events**		HTQ –Part A/1				
**Subjective wellbeing**		WHO-5 Wellbeing index		WHO-5 Wellbeing index	WHO-5 Wellbeing index	WHO-5 Wellbeing index
**Health-related quality of life**		EQ-5D-3L			EQ-5D-3L	EQ-5D-3L
**Self-defined psychosocial goals**		PSYCHLOPS (pre-int. version)		PSYCHLOPS (post-int. version)	PSYCHLOPS (post-int. version)	PSYCHLOPS (post-int. version)
**Post migration stress difficulties**				PMLD	PMLD	PMLD
**Psychological functioning**		WHODAS 2.0		WHODAS 2.0	WHODAS 2.0	WHODAS 2.0
**Cost-effectiveness**		CSSRI-EU			CSSRI-EU	CSSRI-EU
**Adverse events**					**Adverse events form**	

Table 1. Outcome measures and time points

Legend: General Health Questionnaire-12 items (GHQ-12); Mini International Neuropsychiatric Interview (MINI); PTSD Checklist for DSM-V (PCL-5); Patient Health Questionnaire – 9 items (PHQ-9); Harvard Trauma Questionnaire (HTQ); World Health Organization – 5 items (WHO-5) Wellbeing Index; Three-level version of EuroQol Group (EQ-5D-3L); Psychological Outcome Profiles Instrument (PSYCHLOPS); World Health Organization Disability Assessment Schedule (WHODAS); Client Socio-Demographic and Service Receipt Inventory, European Version (CSSRI-EU).

Randomization

Randomization will occur at the individual level and will be stratified by each recruiting centre. To avoid contamination, only one person per household will be randomized. Randomization will be centralized and coordinated by the WHO Collaborating Centre of the University of Verona. Eligible participants will be randomly assigned to one of the two groups with an equal probability of assignment to each group (allocation ratio 1:1). The randomization schedule will be generated using the web-based software Castor Electronic Data Capture[33]. This electronic tool employs a variable block randomization method, in order to allocate groups randomly permuted in blocks of unequal size. The site investigators will not know the block size and will not be able to access the randomization list. The randomization list will be accessible only to the data manager. In addition, the web-based software will allow random allocation only after

For peer review only - http://bmjopen.bmj.com/site/about/guidelines.xhtml

the main information on the enrolled participant is entered, upon verification of the inclusion criteria. After random allocation, the software will produce a unique identification number for each participant. In accordance with the Declaration of Helsinki[34], the participants' confidentiality will be preserved at all times and the contents of the recruitment and follow-up forms will not be disclosed to any third party.

Masking

Masking participants and facilitators about the intervention status will be impossible, due to the nature of the intervention. However, investigators evaluating primary and secondary outcomes post-intervention and at 6- and 12-month follow-up, and the statistician performing all analyses, will be masked to the participants' allocation status. Health care professionals and cultural mediators involved in the assessments will be instructed on how to perform follow-up assessments in order to preserve effective masking.

Sample size and power calculations

On the basis of data extrapolated from prevention trials, an event rate (diagnosis of any mental disorders) of around 15% at six months (primary outcome) is expected[35,36]. However, these prevention trials were conducted in unselected populations that were not exposed to migration stressors. By contrast, refugees and asylum seekers are exposed to stressors that are associated with increased rates of mental disorders[8]. On these grounds, we anticipate an incidence rate of mental disorders of 25% at six months in this population. It is hypothesized that the provision of the SH+ programme will show a clinically significant advantage by producing a between-groups absolute difference of 10%. A sample size of 500 participants per trial, achieves 80% power for a 0.05 level of significance between the two proportions of people diagnosed with mental disorders at six months. Assuming that a relevant proportion of asylum seekers and refugees might be lost at study endpoint (due to the specific characteristics of this population), the final sample size will be of 600 participants (300 in each group) for the European trial and 600 participants (300 in each group) for the Turkish trial. We anticipate that we will screen around 1800-2000 people to have 600 eligible participants.

Adverse events reporting

Serious adverse events and other adverse events reported spontaneously by the participants or observed by the research staff will be recorded on a specifically developed form. Data on the relationship with the study intervention, the action taken regarding intervention, and the outcome of the adverse event, will be collected. An event is considered a potential adverse reaction if it is an undesirable experience occurring to a participant during the study, whether or not it is considered related to the research procedure. This definition includes all aspects of mental health and psychological functioning, but also any other undesirable experiences. The EAB will review spontaneously reported serious adverse reactions (e.g., suicide attempts) within 48h, while general adverse reactions will be reviewed by the EAB in regular meetings.

Data management and statistical analysis

Statistical Analysis

General approach

The statistical analysis will be masked. All analyses will be performed using Stata/SE, Release 14.2.

Two data locks will occur during the study. The first will happen 6 months after the end of the 12-month enrolment period, when information on the primary outcome and on short-term

For peer review only - http://bmjopen.bmj.com/site/about/guidelines.xhtml

secondary outcomes will be available for all the participants. The second will happen 12 months after the end of the 12-month enrolment period. All primary and secondary analyses will be performed on an intention-to-treat (ITT) basis. The ITT population will consist of all participants randomly assigned to the competing intervention strategies, and with data on the baseline assessment available. In order to check the robustness of results, the primary outcome will be additionally analyzed using a per protocol (PP) approach, that will include only SH+ participants who attended at least three sessions. The analysis of the PP population will be used for confirmatory purposes only. If less than 5% of participants do not receive the allocated intervention according to the study protocol, the PP analysis will not be performed. All analyses reaching statistical significance will be replicated for each recruiting centre and for each target language separately.

Analysis of the primary outcome

The proportion of participants with a diagnosis of any mental disorders at 6-months follow-up will be compared between the two groups using a chi-square test (primary analysis). A multivariate analysis (secondary analysis) will be performed through a Poisson regression model, with robust error variance [34] , to estimate relative risks directly and to explore the potential confounding effect of prognostic factors, and the interactions with treatment.

Analysis of the secondary outcomes

The proportion of participants with a diagnosis of any mental disorder post-intervention and at 12-months of follow-up will be compared between the two groups using a chi-square test. A multivariate analysis will be performed using a Poisson regression model, with a robust error variance, to estimate relative risks directly and to explore the potential confounding effect of prognostic factors, and the interactions with treatment[37].

Analysis of GHQ-12, WHODAS 2.0, PHQ-9, WHO-5 Wellbeing index, PSYCHLOPS, PCL-5

For each questionnaire, in case of missing items, the Corrected Item Mean Substitution method[38], will be used (i.e. the item mean across participants weighted by the subject's mean of completed items), using information from subjects belonging to the same treatment arm for the same follow-up time. The hypothesis that the experimental intervention has no effect on GHQ-12, WHODAS 2.0, PHQ-9, WHO-5 Wellbeing index, PSYCHLOPS, PCL-5 scores will be tested by performing seemingly unrelated regression[39], for each time point, controlling for baseline values. In case of joint statistical significance of the coefficients related to treatment status, the effect of treatment on each score will be evaluated through a mixed analysis of covariance (ANCOVA), controlling for the value at baseline. After the last data lock, ANCOVA with robust standard errors will be performed for each scale, using the values at post-intervention, and at 6- and 12-months follow-up, controlling for the value at baseline. In case of joint statistical significance of the coefficients related to treatment status, the analysis will be repeated for each outcome/time combination. Such approaches (assessing the global significance of all time locks for each scale, without imputing missing values) will allow to have a robustness check to results obtained after each time point. As a further sensitivity check, multivariate analyses will be performed for each scale to take confounding factors into account, again including the baseline value as a covariate.

The proportion of participants leaving the study early will be compared between the two groups using a chi-square test (or the Fisher exact test, where appropriate). A multivariate analysis will be performed using a Poisson regression model, with a robust error variance, to estimate

For peer review only - http://bmjopen.bmj.com/site/about/guidelines.xhtml

relative risks directly and to explore the potential confounding effect of prognostic factors, and the interactions with treatment[37].

Cost-effectiveness analysis

Incremental cost-effectiveness analyses (ICEA) will be conducted from the health and social care system perspective and from the societal perspective by means of the net-benefit approach[40]. For the five European countries the ICEA will be conducted from the perspective of the UK health care system and for Turkey from the perspective of the Turkish health care system. Direct costs including formal medical and psychiatric health care, psychosocial care, legal services, and informal services will be used for ICEA from the health and social care system (payer) perspective. Total costs will be used for ICEA from the societal perspective. Comprehensive use of annual health and social care resources will be assessed by means of completing the adapted CSSRI-EU. Information on the unit-costs of the used services for the European countries will be gained by the cost compilation for the UK health and social care system[41]. For Turkey, price lists and other sources will be used to identify unit costs. Costs of informal services will be estimated for both studies on the basis of average salaries per hour. Total direct costs of service use will be calculated by multiplying service units with unit costs. Annual total costs of mental illness will be estimated on the basis of two retrospective assessments of six month service use at six and twelve month follow-up. Productivity losses will be estimated for participants with work permissions by means of the human capital approach[42] on the basis of group-specific average salaries. Costs for the European countries will be calculated in 2017 UK £. Costs for Turkey will be calculated in Turkish Lira (TL). Total costs of health and social care service use will be investigated with regard to the cost driving effects of asylum seekers and refugees characteristics and the impact of different levels of coverage, different macroeconomic, social and health care indicators by estimating regression based cost functions[43]. Incremental NMB will be computed by regressing the NMB on study group[44]. Results of ICEA for the European countries will be interpreted according to UK MWTP thresholds recommended by the NICE[45]. Results for Turkey will be interpreted according to the three times GDP per capita threshold [46] and alternatively by country specific thresholds provided by Woods et al.[47]. For international comparison all cost data and results of ICEA will be converted into € and US$ adjusted for power purchasing parities (PPP).

Patient and Public involvement

Participants have not been involved in the design of the study. However, SH+ facilitators delivering the intervention will be refugees or community members with a refugee or migrant background or with the same/similar culture of participants. They will have some prior experience in health or social or community work or volunteering. The choice of asylum seekers' and refugees' country of origin was made in accordance with a situational analysis on size of refugee populations within European countries and Turkey, as well as feasibility issues.

Ethics and dissemination

It is essential to conduct this research because rigorous evidence needs to be collected on the effectiveness of the SH+ intervention, which has been specifically developed for vulnerable populations. Additionally, it will provide valuable information about optimal adaptation strategies and aspects to consider when scaling up the intervention in diverse contexts.
The trials will be conducted according to globally accepted standards of good clinical practice (as defined in the ICH E6 Guideline for Good Clinical Practice, 1 May 1996), in agreement with the Declaration of Helsinki and in keeping with local regulations.
Dissemination includes a publication plan agreed by all members of the Consortium that regulates academic articles and conference presentations. On the basis of positive results from

For peer review only - http://bmjopen.bmj.com/site/about/guidelines.xhtml

research trials of SH+, the SH+ package will be disseminated by WHO in multiple languages. Any protocol modifications will be submitted to local Ethics Committess for approval.

DISCUSSION

This article presents the main characteristics of two RCTs, which will assess the effectiveness and cost-effectiveness of a low-intensity self help intervention to prevent mental disorders among distressed refugees and asylum seekers. The concept of low-intensity implies that fewer resources are used than in more complex, conventional psychosocial treatments. SH+ may be delivered by facilitators without formal mental health professional training, employs low-cost technologies, such as audio recordings and an illustrated book, has only 5 sessions, and may be delivered to groups of up to 30 participants. The group setting has the advantage of being scalable and optimizing resource use, but it has limited scope for in-depth work with individuals, and participants exposed to traumatic experiences might be concerned about interacting with others in a group space.

If positive results from these trials are achieved, WHO may adopt and disseminate SH+ as a preventive intervention in multiple languages.

A number of characteristics of the design of these two trials may hamper or facilitate their implementation. First, the study was designed as two trials with an almost identical design, with one conducted in European countries and one in Turkey. We reasoned that even though the population that will be recruited in Turkey shares important characteristics with the population in European countries, in terms of pre-migration, migration and post migration stressors[5,9,48], the resettlement context is likely to be different. This may be relevant for a number of aspects, including the definition of the control group, which receives treatment as usual enhanced by referral mechanisms to social organizations and health care facilities. While these mechanisms may show some similarities across different European countries, the same may not apply for Turkey. The resettlement context may additionally be relevant for the capacity of research staff to follow participants, as this aspect may be substantially affected by the mobility of the population under study.

Second, a focus on prevention, rather than treatment, is challenging. In practical terms, it means recruiting participants showing some psychological distress but not a mental disorder. This may represent an obstacle to recruitment, as at study entry the administration of the MINI, which is of paramount relevance to exclude those with a disorder, requires time and training. Additionally, since participants who screen positive cannot be included, we anticipate the need to screen hundreds of potential participants to reach the target sample size. Another aspect related to the recruitment of participants without a disorder is that those eligible for inclusion will clearly be less distressed as compared with those suffering from a mental disorder. Therefore, eligible participants may be less likely to agree to be included, as they may feel that dealing with their psychological distress may not be as relevant as dealing with other social or health aspects. Participants with a mental disorder, by contrast, may recognise that dealing with psychological problems is a high priority and a pre-requisite for optimal social functioning. This may be another obstacle for effective recruitment.

Despite these potential challenges, establishing the effectiveness of preventive psychosocial interventions is a public health priority, as it may help large population groups over short periods of time. Preventive psychosocial interventions must be feasible, sustainable, and cost-

For peer review only - http://bmjopen.bmj.com/site/about/guidelines.xhtml

effective. SH+, which is based on self-help approaches, may have particular advantages for populations with limited access to social and health care services, and with high levels of need.

CONCLUSION

This project aims to generate a strong evidence-base for preventative aspects of SH+, and to create a scientific framework for adapting and equipping social and health care systems in countries inside and outside Europe with such a low intensity intervention. The delivery format of SH+ is innovative, as it requires a short training of non-professional facilitators, and reduced supervision, and fidelity is assured by the pre-recorded audio and the book. Moreover, the intervention has been culturally adapted according to a WHO protocol and cultural adaptation will be extended to other population groups after publication of the SH+ programme.

Authors' contributions

The study was conceived and designed by Mark van Ommeren and Corrado Barbui. Marianna Purgato and Corrado Barbui drafted the manuscript. Ceren Acarturk, Teresa Au, Ken Carswell, Michela Nosè, Giovanni Ostuzzi, Federico Tedeschi, Giulia Turrini, and Mark van Ommeren contributed to the study design and commenting/editing the manuscript drafts.
Sena Akbai, Minna Anttila, Josef Baumgartner, Della Bailey, Massimo Biondi, Martha Bird, Rachel Churchill, Sevde Eskici, Louise Juul Hansen, Paul Heron, Zeynep Ilkkursun, Reinhold Kilian, Markus Koesters, Tella Lantta, Davide Papola, Mariana Popa, Marit Sijbrandij, Lorenzo Tarsitani, Ersin Uygun, Maritta Valimaki, Johannes Wancata, Ross White, Elisa Zanini, Pim Cuijpers provided comments/revisions to the manuscript drafts.

The authors alone are responsible for the views expressed in this article and they do not necessarily represent the views, decisions or policies of the institutions with which they are affiliated.

Acknowledgement

Ethics Advisory Board members: Anna Chiumento, Stefan Weinmann

Funding statement

This work is supported by the European Commission, grant agreement n. 779255 "RE-DEFINE: Refugee Emergency: DEFining and Implementing Novel Evidence-based psychosocial interventions".

The funder had no role in study design, and will have no role in data management, analysis, interpretation of data, writing the report and the decision to submit the report for publication, nor ultimate authority over any of the listed activities. EU will have the possibility to audit the financial project management, while for the scientific part deliverables and milestones are being provided to the EU according to the grant agreement.

Competing interests statement

None.

For peer review only

For peer review only - http://bmjopen.bmj.com/site/about/guidelines.xhtml

Figure legend

Figure 1. RCT Flow diagram

For peer review only

For peer review only - http://bmjopen.bmj.com/site/about/guidelines.xhtml

References

1. UNHCR. Global trends. Forced displacement in 2017. Available from https://www.unhcr.org/globaltrends2017/ Accessed March 6, 2019.

2. UNHCR. Global trends. Forced displacement in 2015. Availeble from https://www.unhcr.org/statistics/unhcrstats/576408cd7/unhcr-global-trends-2015.html Accessed March 6, 2019.

3. UNHCR. Global trends. Forced displacement in 2016. Available from https://www.unhcr.org/statistics/unhcrstats/5943e8a34/global-trends-forced-displacement-2016.html Accessed March 6, 2019.

4. Wells R, Steel Z, Abo-Hilal M, Hassan AH, Lawsin C. Psychosocial concerns reported by Syrian refugees living in Jordan: systematic review of unpublished needs assessments. *Br J Psychiatry.* 2016;209(2):99-106.

5. Kirmayer LJ, Narasiah L, Munoz M, et al. Common mental health problems in immigrants and refugees: general approach in primary care. *CMAJ.* 2011;183(12):E959-E967.

6. Miller KE, Rasmussen A. The mental health of civilians displaced by armed conflict: an ecological model of refugee distress. *Epidemiol Psychiatr Sci.* 2016:1-10.

7. World Health Organization Regional Office for Europe. Mental health promotion and mental health care in refugees and migrants. Technical guidance. Copenhagen:WHO;2018.

8. Priebe S, D G, R E-N. Public health aspects of mental health among migrants and refugees: a review of the evidence on mental health care for refugees, asylum seekers and irregular migrants in the WHO European Region (2016). In:2016.

9. Miller KE, Rasmussen A. War exposure, daily stressors, and mental health in conflict and post-conflict settings: bridging the divide between trauma-focused and psychosocial frameworks. *Soc Sci Med.* 2010;70(1):7-16.

10. Alemi Q, Stempel C. Discrimination and distress among Afghan refugees in northern California: The moderating role of pre- and post-migration factors. *PLoS One.* 2018;13(5):e0196822.

11. Purgato M, Tol WA, Bass JK. An ecological model for refugee mental health: implications for research. *Epidemiol Psychiatr Sci.* 2016:1-3.

12. Turrini G, Purgato M, Ballette F, Nose M, Ostuzzi G, Barbui C. Common mental disorders in asylum seekers and refugees: umbrella review of prevalence and intervention studies. *Int J Ment Health Syst.* 2017;11:51.

13. Zhong BL, Liu TB, Chan SSM, et al. Common mental health problems in rural-to-urban migrant workers in Shenzhen, China: prevalence and risk factors. *Epidemiol Psychiatr Sci.* 2018;27(3):256-265.

For peer review only - http://bmjopen.bmj.com/site/about/guidelines.xhtml

14. Nose M, Ballette F, Bighelli I, et al. Psychosocial interventions for post-traumatic stress disorder in refugees and asylum seekers resettled in high-income countries: Systematic review and meta-analysis. *PLoS One.* 2017;12(2):e0171030.

15. Giacco D, Laxhman N, Priebe S. Prevalence of and risk factors for mental disorders in refugees. *Semin Cell Dev Biol.* 2017.

16. Giacco D, Priebe S. Mental health care for adult refugees in high-income countries. *Epidemiol Psychiatr Sci.* 2017:1-8.

17. G. Turrini MP, C. Acarturk, M. Anttila, T. Au, F. Ballette, M. Bird, K. Carswell, R. Churchill, P. Cuijpers, J. Hall, L. J. Hansen, M. Kösters, T. Lantta, M. Nosè, G. Ostuzzi, M. Sijbrandij, F. Tedeschi, M. Valimaki, J. Wancata, R. White, M. van Ommeren, C. Barbui. Efficacy and acceptability of psychosocial interventions in asylum seekers and refugees: systematic review and meta-analysis. *Epidemiol Psychiatr Sci.* in press.

18. Epping-Jordan JE, Harris R, Brown FL, et al. Self-Help Plus (SH+): a new WHO stress management package. *World Psychiatry.* 2016;15(3):295-296.

19. Tol WA, Augustinavicius J, Carswell K, et al. Translation, adaptation, and pilot of a guided self-help intervention to reduce psychological distress in South Sudanese refugees in Uganda. *Glob Ment Health (Camb).* 2018;5:e25.

20. Tol WA, Augustinavicius J, Carswell K, et al. Feasibility of a guided self-help intervention to reduce psychological distress in South Sudanese refugee women in Uganda. *World Psychiatry.* 2018;17(2):234-235.

21. Goldberg DP, Blackwell B. Psychiatric illness in general practice. A detailed study using a new method of case identification. *Br Med J.* 1970;1(5707):439-443.

22. Sheehan DV, Lecrubier Y, Sheehan KH, et al. The Mini-International Neuropsychiatric Interview (M.I.N.I.): the development and validation of a structured diagnostic psychiatric interview for DSM-IV and ICD-10. *J Clin Psychiatry.* 1998;59 Suppl 20:22-33.

23. American Psychiatric Association. The Diagnostic and Statistical Manual of Mental Disorders (5th ed.; DSM–5). Washington DC: American Psychiatric Association; 2013.

24. World Health Organization, Geneva. The ICD-10 Classification of mental and behavioural disorders: clinical descriptions and diagnostic guidelines. 1992.

25. Begg C, Cho M, Eastwood S, et al. Improving the quality of reporting of randomized controlled trials. The CONSORT statement. *JAMA.* 1996;276(8):637-639.

26. Schulz KF, Altman DG, Moher D. CONSORT 2010 statement: Updated guidelines for reporting parallel group randomised trials. *J Pharmacol Pharmacother.* 2010;1(2):100-107.

For peer review only - http://bmjopen.bmj.com/site/about/guidelines.xhtml

27. Cuijpers P, Munoz RF, Clarke GN, Lewinsohn PM. Psychoeducational treatment and prevention of depression: the "Coping with Depression" course thirty years later. *Clin Psychol Rev.* 2009;29(5):449-458.

28. den Boer PC, Wiersma D, Van den Bosch RJ. Why is self-help neglected in the treatment of emotional disorders? A meta-analysis. *Psychol Med.* 2004;34(6):959-971.

29. World Health Organization, Geneva. Intervention Guide for Mental, Neurological and Substance Use Disorders in Non-Specialized Health Settings: Mental Health Gap Action Programme (mhGAP). WHO:Geneva;2010.

30. Hayes SC, Levin ME, Plumb-Vilardaga J, Villatte JL, Pistorello J. Acceptance and commitment therapy and contextual behavioral science: examining the progress of a distinctive model of behavioral and cognitive therapy. *Behav Ther.* 2013;44(2):180-198.

31. Tjak JG, Davis ML, Morina N, Powers MB, Smits JA, Emmelkamp PM. A meta-analysis of the efficacy of acceptance and commitment therapy for clinically relevant mental and physical health problems. *Psychother Psychosom.* 2015;84(1):30-36.

32. Fledderus M, Bohlmeijer ET, Pieterse ME, Schreurs KM. Acceptance and commitment therapy as guided self-help for psychological distress and positive mental health: a randomized controlled trial. *Psychol Med.* 2012;42(3):485-495.

33. Castor Electronic Data Capture, Ciwit BV, Amsterdam, The Netherlands, 2018.

34. World Medical Association. World Medical Association Declaration of Helsinki: ethical principles for medical research involving human subjects. *JAMA* 2013;310:2191-2194.

35. Cuijpers P, van SA, Smit F, Mihalopoulos C, Beekman A. Preventing the onset of depressive disorders: a meta-analytic review of psychological interventions. *Am J Psychiatry.* 2008;165(10):1272-1280.

36. van ZK, Buntrock C, Ebert DD, et al. Preventing the onset of major depressive disorder: a meta-analytic review of psychological interventions. *Int J Epidemiol.* 2014;43(2):318-329.

37. Zou G. A modified poisson regression approach to prospective studies with binary data. *Am J Epidemiol.* 2004;159(7):702-706.

38. JME H. *Item nonresponse: occurrence, causes, and imputation of missing answers to test items.* Leiden: DSWO Press; 1999.

39. A Z. An Efficient Method of Estimating Seemingly Unrelated Regressions and Tests for Aggregation Bias. *Journal of the American Statistical Association,.* 1962;57(298):348-368.

40. Glick H. Economic evaluation in clinical trials (Reprinted.). In: Press OU, ed. *Handbooks in Health Economic Evaluation Series.*2010.

For peer review only - http://bmjopen.bmj.com/site/about/guidelines.xhtml

41. Curtis L, & Burns, A. *Unit Costs of Health and Social Care 2017.* 2017.

42. Brouwer WB, Koopmanschap MA. How to calculate indirect costs in economic evaluations. *Pharmacoeconomics.* 1998;13(5 Pt 1):563-569.

43. Knapp M. Making music out of noise--the cost function approach to evaluation. *Br J Psychiatry Suppl.* 1998(36):7-11.

44. Hoch JS, Briggs, A. H., & Willan, R. Something old, something new, something borrowed, something blue: a framework for the marriage of health econometrics and cost-effectiveness analysis. *Health Econ.* 2002;11:415-430.

45. Appleby J. Crossing the line: NICE's value for money threshold. *BMJ.* 2016;352:i1336.

46. Robinson LA, Hammitt, J. K., Chang, A. Y., & Resch, S. . Understanding and improving the one and three times GDP per capita cost-effectiveness thresholds. *Health Policy And Planning.* 2017;32:141-145.

47. Woods B, Revill, P., Sculpher, M., & Claxton, K. Country-Level Cost-Effectiveness Thresholds: Initial Estimates and the Need for Further Research. *Value in Health : the Journal of the International Society for Pharmacoeconomics and Outcomes Research.* 2016;19:929-935.

48. Sijbrandij M, Acarturk C, Bird M, et al. Strengthening mental health care systems for Syrian refugees in Europe and the Middle East: integrating scalable psychological interventions in eight countries. *Eur J Psychotraumatol.* 2017;8(sup2):1388102.

For peer review only

For peer review only - http://bmjopen.bmj.com/site/about/guidelines.xhtml

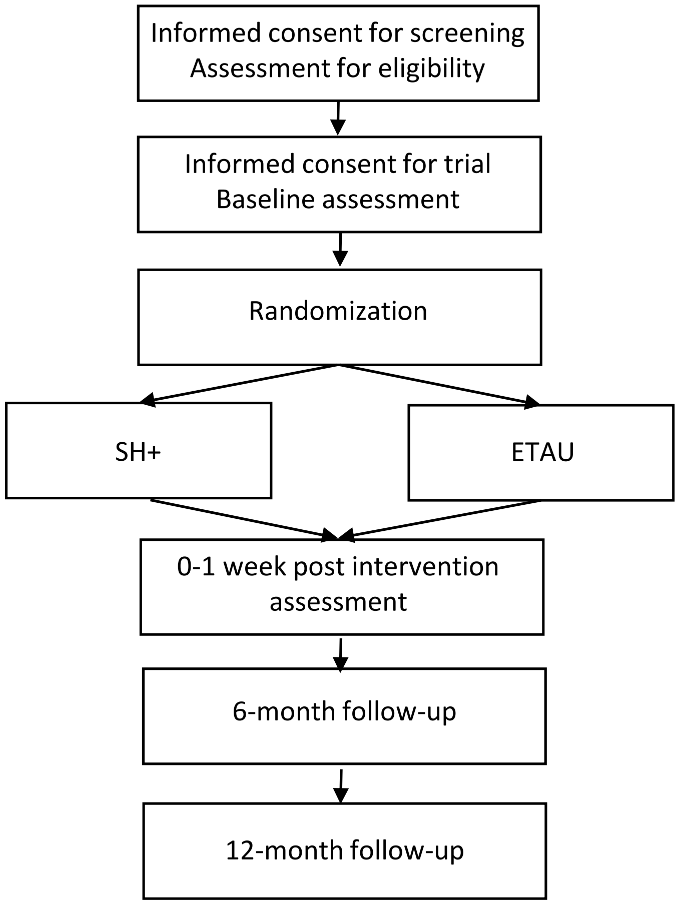

Figure 1. RCT Flow diagram

SPIRIT

STANDARD PROTOCOL ITEMS: RECOMMENDATIONS FOR INTERVENTIONAL TRIALS

SPIRIT 2013 Checklist: Recommended items to address in a clinical trial protocol and related documents*

Section/item	Item No	Description	pages
**Administrative information**			
Title	1	Descriptive title identifying the study design, population, interventions, and, if applicable, trial acronym	1
Trial registration	2a	Trial identifier and registry name. If not yet registered, name of intended registry	3
	2b	All items from the World Health Organization Trial Registration Data Set	3
Protocol version	3	Date and version identifier	3
Funding	4	Sources and types of financial, material, and other support	13
Roles and responsibilities	5a	Names, affiliations, and roles of protocol contributors	1-2
	5b	Name and contact information for the trial sponsor	13
	5c	Role of study sponsor and funders, if any, in study design; collection, management, analysis, and interpretation of data; writing of the report; and the decision to submit the report for publication, including whether they will have ultimate authority over any of these activities	13
	5d	Composition, roles, and responsibilities of the coordinating centre, steering committee, endpoint adjudication committee, data management team, and other individuals or groups overseeing the trial, if applicable (see Item 21a for data monitoring committee)	13
**Introduction**			
Background and rationale	6a	Description of research question and justification for undertaking the trial, including summary of relevant studies (published and unpublished) examining benefits and harms for each intervention	4-5
	6b	Explanation for choice of comparators	7
Objectives	7	Specific objectives or hypotheses	
Trial design	8	Description of trial design including type of trial (eg, parallel group, crossover, factorial, single group), allocation ratio, and framework (eg, superiority, equivalence, noninferiority, exploratory)	4-5
**Methods: Participants, interventions, and outcomes**			**4-5**
Study setting	9	Description of study settings (eg, community clinic, academic hospital) and list of countries where data will be collected.	4

For peer review only

		Reference to where list of study sites can be obtained	
Eligibility criteria	10	Inclusion and exclusion criteria for participants. If applicable, eligibility criteria for study centres and individuals who will perform the interventions (eg, surgeons, psychotherapists)	5
Interventions	11a	Interventions for each group with sufficient detail to allow replication, including how and when they will be administered	6-7; Figure 1
	11b	Criteria for discontinuing or modifying allocated interventions for a given trial participant (eg, drug dose change in response to harms, participant request, or improving/worsening disease)	9
	11c	Strategies to improve adherence to intervention protocols, and any procedures for monitoring adherence (eg, drug tablet return, laboratory tests)	NA
	11d	Relevant concomitant care and interventions that are permitted or prohibited during the trial	-
Outcomes	12	Primary, secondary, and other outcomes, including the specific measurement variable (eg, systolic blood pressure), analysis metric (eg, change from baseline, final value, time to event), method of aggregation (eg, median, proportion), and time point for each outcome. Explanation of the clinical relevance of chosen efficacy and harm outcomes is strongly recommended	7; Table1
Participant timeline	13	Time schedule of enrolment, interventions (including any run-ins and washouts), assessments, and visits for participants. A schematic diagram is highly recommended (see Figure)	Figure 1
Sample size	14	Estimated number of participants needed to achieve study objectives and how it was determined, including clinical and statistical assumptions supporting any sample size calculations	9
Recruitment	15	Strategies for achieving adequate participant enrolment to reach target sample size	9
**Methods: Assignment of interventions (for controlled trials)**			
Allocation:			
Sequence generation	16a	Method of generating the allocation sequence (eg, computer-generated random numbers), and list of any factors for stratification. To reduce predictability of a random sequence, details of any planned restriction (eg, blocking) should be provided in a separate document that is unavailable to those who enrol participants or assign interventions	8
Allocation concealment mechanism	16b	Mechanism of implementing the allocation sequence (eg, central telephone; sequentially numbered, opaque, sealed envelopes), describing any steps to conceal the sequence until interventions are assigned	8
Implementation	16c	Who will generate the allocation sequence, who will enrol participants, and who will assign participants to interventions	8
Blinding (masking)	17a	Who will be blinded after assignment to interventions (eg, trial participants, care providers, outcome assessors, data analysts), and how	9
	17b	If blinded, circumstances under which unblinding is permissible,	9

For peer review only

		and procedure for revealing a participant's allocated intervention during the trial	
**Methods: Data collection, management, and analysis**			
Data collection methods	18a	Plans for assessment and collection of outcome, baseline, and other trial data, including any related processes to promote data quality (eg, duplicate measurements, training of assessors) and a description of study instruments (eg, questionnaires, laboratory tests) along with their reliability and validity, if known. Reference to where data collection forms can be found, if not in the protocol	Table 1
	18b	Plans to promote participant retention and complete follow-up, including list of any outcome data to be collected for participants who discontinue or deviate from intervention protocols	NA
Data management	19	Plans for data entry, coding, security, and storage, including any related processes to promote data quality (eg, double data entry; range checks for data values). Reference to where details of data management procedures can be found, if not in the protocol	9-10
Statistical methods	20a	Statistical methods for analysing primary and secondary outcomes. Reference to where other details of the statistical analysis plan can be found, if not in the protocol	9-11
	20b	Methods for any additional analyses (eg, subgroup and adjusted analyses)	10
	20c	Definition of analysis population relating to protocol non-adherence (eg, as randomised analysis), and any statistical methods to handle missing data (eg, multiple imputation)	10
**Methods: Monitoring**			
Data monitoring	21a	Composition of data monitoring committee (DMC); summary of its role and reporting structure; statement of whether it is independent from the sponsor and competing interests; and reference to where further details about its charter can be found, if not in the protocol. Alternatively, an explanation of why a DMC is not needed	6
	21b	Description of any interim analyses and stopping guidelines, including who will have access to these interim results and make the final decision to terminate the trial	9-11
Harms	22	Plans for collecting, assessing, reporting, and managing solicited and spontaneously reported adverse events and other unintended effects of trial interventions or trial conduct	9
Auditing	23	Frequency and procedures for auditing trial conduct, if any, and whether the process will be independent from investigators and the sponsor	13
**Ethics and dissemination**			
Research ethics approval	24	Plans for seeking research ethics committee/institutional review board (REC/IRB) approval	11 (already approved in all sites)

For peer review only

Protocol amendments	25	Plans for communicating important protocol modifications (eg, changes to eligibility criteria, outcomes, analyses) to relevant parties (eg, investigators, REC/IRBs, trial participants, trial registries, journals, regulators)	12
Consent or assent	26a	Who will obtain informed consent or assent from potential trial participants or authorised surrogates, and how (see Item 32)	-
	26b	Additional consent provisions for collection and use of participant data and biological specimens in ancillary studies, if applicable	NA
Confidentiality	27	How personal information about potential and enrolled participants will be collected, shared, and maintained in order to protect confidentiality before, during, and after the trial	8-9
Declaration of interests	28	Financial and other competing interests for principal investigators for the overall trial and each study site	13
Access to data	29	Statement of who will have access to the final trial dataset, and disclosure of contractual agreements that limit such access for investigators	-
Ancillary and post-trial care	30	Provisions, if any, for ancillary and post-trial care, and for compensation to those who suffer harm from trial participation	NA
Dissemination policy	31a	Plans for investigators and sponsor to communicate trial results to participants, healthcare professionals, the public, and other relevant groups (eg, via publication, reporting in results databases, or other data sharing arrangements), including any publication restrictions	11
	31b	Authorship eligibility guidelines and any intended use of professional writers	13
	31c	Plans, if any, for granting public access to the full protocol, participant-level dataset, and statistical code	11
**Appendices**			
Informed consent materials	32	Model consent form and other related documentation given to participants and authorised surrogates	-
Biological specimens	33	Plans for collection, laboratory evaluation, and storage of biological specimens for genetic or molecular analysis in the current trial and for future use in ancillary studies, if applicable	NA

*It is strongly recommended that this checklist be read in conjunction with the SPIRIT 2013 Explanation & Elaboration for important clarification on the items. Amendments to the protocol should be tracked and dated. The SPIRIT checklist is copyrighted by the SPIRIT Group under the Creative Commons "Attribution-NonCommercial-NoDerivs 3.0 Unported" license.

For peer review only - http://bmjopen.bmj.com/site/about/guidelines.xhtml
