# Supplementary material for: Effectiveness and cost-effectiveness of Self-Help Plus (SH+) for preventing mental disorders in refugees and asylum seekers in Europe and Turkey: study protocols for two randomised controlled trials
Source: BMJ Open. 2019 May 14;9(5):e030259. doi: 10.1136/bmjopen-2019-030259 (PMC6530324; doi:10.1136/bmjopen-2019-030259)
Supplement: Reviewer comments [file bmjopen-2019-030259.reviewer_comments.pdf]

PEER REVIEW HISTORY

BMJ Open publishes all reviews undertaken for accepted manuscripts. Reviewers are asked to complete a checklist review form (**http://bmjopen.bmj.com/site/about/resources/checklist.pdf**) and are provided with free text boxes to elaborate on their assessment. These free text comments are reproduced below.

ARTICLE DETAILS

**TITLE (PROVISIONAL)**	Effectiveness and cost-effectiveness of Self Help Plus (SH+) for preventing mental disorders in refugees and asylum seekers in Europe and Turkey: study protocols for two randomized controlled trials
**AUTHORS**	Purgato, Marianna; Carswell, Kenneth; Acarturk, Ceren; Au, Teresa; Akbai, Sena; Anttila, Minna; Baumgartner, Josef; Bailey, Della; Biondi, Massimo; Bird, Martha; Churchill, R; Eskici, Sevde; Hansen, Louise; Heron, Paul; Ilkkursun, Zeynep; Kilian, Reinhold; Koesters, Markus; Lantta, Tella; Nosè, Michela; Ostuzzi, Giovanni; Papola, Davide; Popa, Mariana; Sijbrandij, Marit; Tarsitani, Lorenzo; Tedeschi, Federico; Turrini, Giulia; Uygun, Ersin; Välimäki, Maritta; Wancata, Johannes; White, Ross; Zanini, Elisa; Cuijpers, Pim; Barbui, Corrado; Van Ommeren, Mark

This article was not externally reviewed at BMJ Open. Protocols that have been independently assessed prior to submission to BMJ Open are usually fast-tracked to publication on the grounds that further substantial changes will not be possible. This independent assessment will usually be external, independent review for both a substantial grant award from a non-commercial or government funder and ethics approval.
